# From fossil trader to paleontologist: on Swiss-born naturalist Santiago Roth and his scientific contributions

**DOI:** 10.1186/s13358-023-00282-6

**Published:** 2023-09-11

**Authors:** Marcelo R. Sánchez-Villagra, Mariano Bond, Marcelo Reguero, Tomás Bartoletti

**Affiliations:** 1 Department of Paleontology, University of Zurich, Karl-Schmid-Straße 4, 8006 Zurich, Switzerland; 2 Facultad de Ciencias Naturales Y Museo, Universidad Nacional de La Plata, CONICET (Consejo Nacional de Investigaciones, Científicas Y Técnicas, Buenos Aires, Argentina; 3 Max Weber Postdoctoral Programme, European University Institute, Florence, Italy

**Keywords:** History, Exploration, Zurich, Taxonomy, Geology, Ameghino, Notoungulata, Pampa, Historia, Exploración, Zurich, Taxonomía, Geología, Ameghino, Notoungulata, Pampa

## Abstract

Roth’s explorations, the resulting collections many now allocated in La Plata, Zurich, Geneva and Copenhagen, and his significant contributions in geological—especially stratigraphic—and paleontological topics, are a paradigmatic case for the global history of paleontology and for the Swiss migration history in Latin America. His work included the discovery of a diverse megafauna from the Pampean region, of sites and strata in Patagonia of paleontological significance, and the recognition of a group of endemic ungulate mammals, Notoungulata. Roth’s discovery of a human skeleton associated with a glyptodont carapace is one of the first reports of the coexistence of humans with the extinct fauna of the South American Quaternary. Roth became a renowned scholar at the Museo de La Plata, which was a leading scientific institution in the nation-making of Argentina, particularly in the expansion of the Patagonian frontier. He also kept strong ties with his native Switzerland, where late in his adult life he obtained some formal training and tried to attract other Swiss nationals to work in natural sciences in Argentina. His biography sheds light about the circumstances of his scientific collection and career in the interstices between amateur and professional science, modernity and imperialism at the turn of the twentieth century.

**Supplementary Information:**

The online version contains supplementary material available at 10.1186/s13358-023-00282-6.

## Resumen

Las exploraciones de Roth, las colecciones resultantes muchas ahora ubicadas en La Plata, Zúrich, Ginebra y Copenhague, y sus importantes aportes en temas geológicos -especialmente estratigráficos- y paleontológicos, son un caso paradigmático para la historia global de la paleontología y para la historia de la migración suiza en América Latina. Su trabajo exploratorio incluyó el descubrimiento de una megafauna diversa de la región pampeana, de sitios y estratos en la Patagonia de importancia paleontológica, y el reconocimiento de un grupo de mamíferos ungulados endémicos, Notoungulata. El descubrimiento de Roth de un esqueleto humano asociado a un caparazón de gliptodonte es uno de los primeros informes de la coexistencia del hombre y la fauna extinta del Cuaternario sudamericano. Roth se convirtió en un académico de renombre en el Museo de La Plata, que fue una institución científica líder en la formación de la nación argentina, particularmente en la expansión de la frontera patagónica. También mantuvo fuertes lazos con su Suiza natal, donde al final de su vida adulta obtuvo una formación formal y trató de atraer a otros ciudadanos suizos para trabajar en ciencias naturales en Argentina. Su biografía arroja luz sobre las circunstancias de su acervo científico y su trayectoria en los intersticios entre la ciencia amateur y la profesional, la modernidad y el imperialismo a principios del siglo XX.

## Introduction

**Fig. 1:**
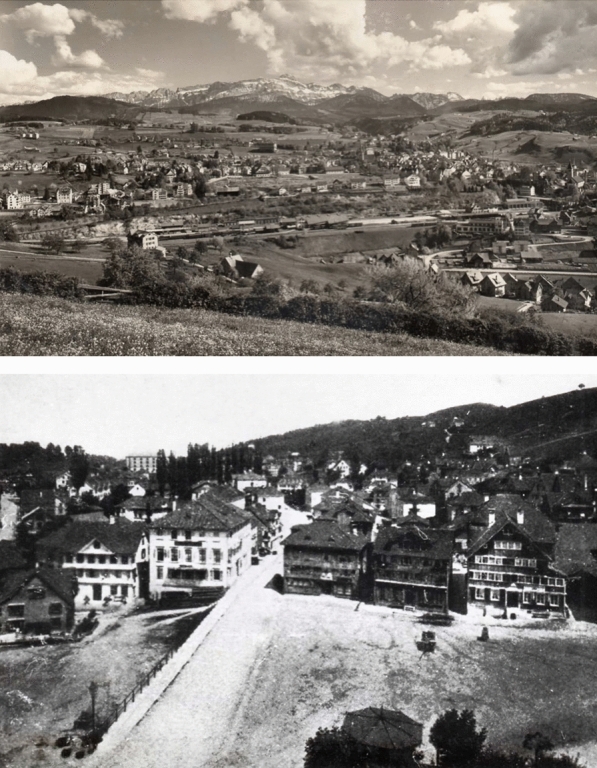
Top: view of Herisau with the “Säntis” mountain in the background. Bottom: the ‘Obstmarkt’ in Herisau around 1945. Roth’s birthplace was one of the houses by the Markt. Pictures from archives at the Museo de La Plata

The life of Santiago Roth, born Kaspar Jakob Roth-Schuetz on June 14, 1850 in Herisau ‘am Obstmarkt’, Appenzell, Switzerland (Fig. 1), exemplifies the experience of many Swiss families during the nineteenth century that were marked by transatlantic migration. As an introduction to this special issue with original research contributions on fossils collected by the late Santiago Roth, we provide a historical overview on his life and works, of marked influence in Argentinian paleontology and the establishment of museum collections at both sides of the Atlantic. Much of this biographical account is based on obituaries and recent articles about the history of paleontology in Argentina, as cited below, as well as archival work.

## Family, migration and first collections

As the eldest of 12 children, Kaspar Jakob was the son of a Bernese farmer, Johann Jakob Roth, and his mother, Ursula Tobler, who was a relative of the linguist Tobler of the family of pastors that had already been thriving in the seventeenth century and bore many famous pulpit orators (Weigelt, 1951). Roth’s family settled in 1860 in the nearby city of St. Gallen. In addition to managing a small farm, father Roth seems to have also operated wainwright business, i.e., making and repairing wagons and carts. Apparently for economic reasons, in 1866 the whole Roth family emigrated to Argentina, a promised land for many Swiss at the time.

The Roth family settled in the town of Baradero in the Buenos Aires province, where settlement of Swiss immigrants had begun in 1856, mainly by the efforts of authorities, such as Martín de Gainza and the German educator German Frers (Schobinger, 1957). The once called ‘Schweizerhaus’, the Swiss house, would be used for meetings and is still known as ‘Casa suiza’. By the time of emigration, Jakob Roth was only 16 years. He then started using the Spanish version of his second name, Santiago, and following the advice of his father, he took up the trade of saddler, fitting for the country. When Roth moved to Pergamino, also in the province of Buenos Aires, where he worked as a saddler, he began to collect naturalia and fossils in the Quaternary sediments of the same Province, in what became known as the Pampean Formation (Voglino et al., 2023).

During the period of Roth’s migration, Argentina became a consolidated nation, as Buenos Aires as a state rejoined the rest of the Provinces forming the Argentine Confederation or Republic. Although engaged in provincial feuds and in an international war (the Paraguayan war) the policy to attract and integrate European migrants was established after Rosas fell in 1852. Regions such as the Pampas and Patagonia were part of the capitalist expansion of the Argentinian state, which involved dispossession of indigenous lands, military occupation, and projects of settler colonialism (Larson, 2020). The lands in Buenos and Santa Fé Provinces where many of the Swiss migrants settled where lands that had been occupied early since the Spanish conquest, whereas occupation of South border’s lands began only as a final push in 1879 (Viñas, 1982).

Among other Europeans, Swiss migrants were welcomed as ‘modern’ workers with Protestant ethos that forged a more productive land management and exploitation of natural resources (Karrer, 1888–1889; Schobinger, 1957). Santiago Roth could make use of his learned skills both as saddler and ‘amateur’ collector. It was already in his school times in Switzerland, where the popularization of science triggered his interest in the collection of natural objects. While attending school, he became acquainted with Bernhard Wartmann, director of the local St. Gallen Museum, who encouraged his natural history lessons and taught him the collection and preparation of plants and animals. Even before Argentina, Roth at 16 years of age possessed a comprehensive herbarium, a butterfly collection and a sizeable collection of minerals and stones (Machon, 1925; Weigelt, 1951).

Roth´s passage from being an ‘amateur’ collector to a professional scientist should be framed in the context of the commodification of natural objects and fossils in this period globally, and particularly in the field of paleontology in Argentina. In the nineteenth century, the sale of natural science specimens was a way to earn a living (Podgorny, 2001, 2002, 2012), as exemplified also by the most influential and celebrated of paleontologists in Argentina, Florentino Ameghino (Boscaini et al., 2021; Casinos, 2012; Simpson, 1984). With connections in museums of European cities, fossils could be sold in good prices. Migrants such as Roth were not only artisans, peasant or workers, but generally entrepreneurs. The fossil collections offered Roth an additional option for financial sustainability. For example, since his arrival in Baradero, in addition to his tasks as a saddler, Roth was commissioned by various museums in Switzerland (including the one in St. Gallen) and began to tour the countryside collecting plants and animals that he then sent to such institutions. As Weigelt (1951) reported, Roth undertook with little means an adventurous sailing trip on the Paraná with two young Swiss individuals. One of them, a clockmaker, earned the necessary money for maintenance through ambulatory clock repairs. Probably difficulties related to the Paraguay war forced the young traveler to return home.

In 1876, while searching fossils with his friend José Mayorotti, Roth found remains of a human skeleton near Pergamino, the ‘Saladero’ skeleton, which became one of the first reputedly human skeletons found in the ‘Pampean’ Pleistocene together with the earlier finds of Seguin (Carcarañá skeleton) and Ameghino (Arroyo Frías skeleton) (Hrdlička et al., 1912). The ‘Saladero’ skeleton remains were brought to the Museo Público of Buenos Aires, and G. Burmeister kept them, but he was not convinced that they were ancient or Pleistocene fossils (Burmeister, 1879). G. Burmeister (1807–1892) was the Director of the National Museum of Buenos Aires, a German born migrant who adopted Argentina as his new home. When Roth presented the specimen from Pontimelo, in words of Roth, Burmeister said to him that this and the Saladero specimens belonged to the Pampean Formation (Pleistocene), but in his works Burmeister doubted this assessment (see Roth, 1888 and Hrdlička et al., 1912), an example of how the evidence of fossil men in the pampas was not easily accepted by some of the established scientists at the time (Lehmann Nitzsche, 1907).

In 1877 or 1878 Roth sold his first fossil collection to a wealthy Dane living in Buenos Aires, Dr. Valdemar Lausen (1834–1889), a medical doctor who worked and lived for most of his adult life in Buenos Aires. Lausen donated it to the Copenhagen Zoological Museum (Hansen, 2020). From Roth, Lausen bought everything in catalogues N° 2 and N° 3 (Roth, 1882). These fossil mammals were gathered from different localities of the Northwest of Buenos Aires Province (Arroyo Ramallo, Arroyo Pergamino, Arroyo del Medio) and Entre Ríos Province (Paraná River). The large V. Lausen Collection holds at least 55 mammalian species. The represented species are relatively diverse, ranging from aquatic mammals such as dolphins and seals to sabre-toothed cats and enormous ground sloths (Hansen, 2020; Winge, 1888). Some partial skeletons of Xenartha of the Lausen collection are exhibited in the Natural History Museum of Denmark, i.e., *Megatherium* and *Glyptodon*.

The Lausen collection of fossils collected by Roth in Copenhagen is also important for the historical taxonomy of fossil mammal faunas, as it contains several holotypes of species described by Florentino Ameghino: the interatheriid notoungulate *Protypotherium antiquum* Ameghino, 1882, the litoptern *Scalabrinitherium rothii* Ameghino, 1882, and the capybara-like *Neoprocavia mesopotamica* Ameghino, 1889 (Additional file 1). Roth’s specimen *Protypotherium antiquum* was further described by Ameghino (1889) and even illustrated in his Atlas (Ameghino, 1889, plate 15: Fig. 1). Roth kept this specimen until he sold it to V. Lausen. He had collected it in the cliffs of Paraná River (Entre Ríos Province) probably during an excursion in 1881; later Ameghino borrowed it from Roth for its study and the description of the dental morphology of the species (Ameghino, 1885, 1889). This specimen has a label handwritten by Roth with the following information: ‘*Protypotherium antiquum* Amegh. Delta-Egnen, Entre Ríos. Original Roth (37.), Lausen. 25.11.87. 208*’ (Winge, 1888). The term ‘Original Roth (37.)’ refers to the number in Roth’s catalogue, and 208 refers to an older catalogue number of Museum of Zoology in Copenhagen (NHMD ZMK 208). Nowadays, this material is catalogued as NHMD ZMK 21/1887 (Fernández et al., 2018).

The zoological museum in Copenhagen bought another collection from Roth directly in 1883 or 1884 (Hansen, 2019; Weigelt, 1951). This collection includes human remains known as ‘Hombre de Pontimelos or Fontezuelas’, discussed below.

Roth collection activities intensified after the dealings with V. Lausen. He was inspired by the work of G. Burmeister. Following Burmeister’s advice, Roth began to collect fossil remains of various Quaternary mammals, as well as specimens of current fauna and flora. The fossils, extremely abundant in diverse sites of the Argentinian Pampas, were of special interest to foreign museums. Roth became not only an excellent collector of fossils, but also a keen observer of the geology and stratigraphy of the Pampas (Roth, 1882, 1888, 1889a).

In addition to his fossil business opening its trade networks to Europe and beyond, he became more refined in his collections. Arguably, his beginning as ‘amateur’ scientist was grounded on the extremely valuable cognizance of the territory. The knowledge of the field and on the ground were, indeed, more and more required to improve not only the amount of collected fossils but also for more precise data collection. As explained below, Roth´s trip to his homeland, Switzerland, allowed him to receive academic training and eventually an honorary doctorate from the University of Zurich. This new condition as ‘scientific’ collector facilitated the expansion of his fossils business, and he was offered an academic position at the Museo de La Plata. Roth’s biography is a singular case of migration and entrepreneurship in the history of science between Switzerland and the Argentinian Pampas.

## Becoming a paleontologist: Roth’s return to Switzerland

**Fig. 2:**
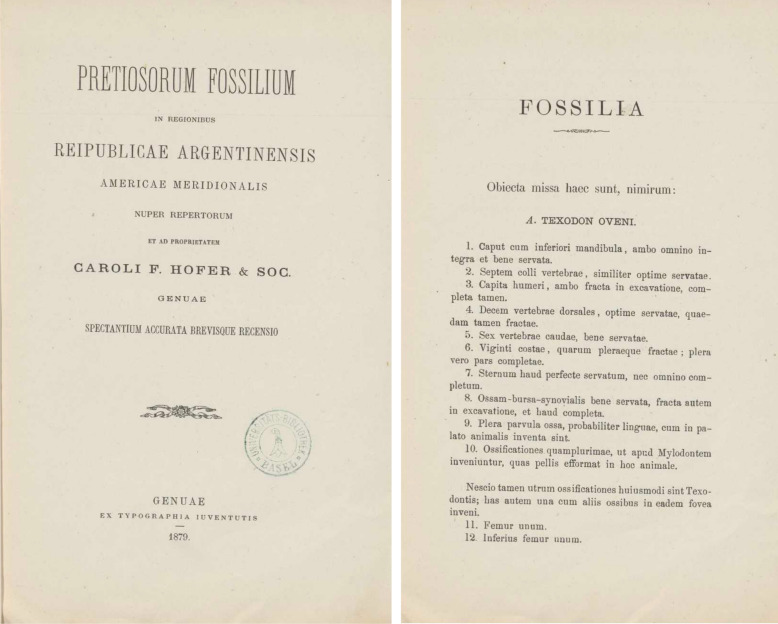
First descriptive catalogue of specimens collected by Santiago Roth offered for sale. It was drafted in Latin and titled in the name of the commissioner ‘Carolus F. Hofer’, Roth’s brother in law, without naming Roth

By the late 1870s, Roth’s expanding collections needed to find more suitable buyers in Europe. Roth believed Paris could be an excellent client, one with capable workers for restauration and preparation (Argot, 2008). A partnership with his brother-in-law Carlo Hofer, who lived in Genoa, provided the basis required to ship and trade with European museums. In 1879 they managed to gather a new collection and published a first descriptive catalogue of these specimens (Fig. 2), which was drafted in Latin and titled in the name of the commissioner ‘Carolus F. Hofer’, without naming Roth. It was not in Paris but in Geneva that the prominent German physician and naturalist Carl Vogt, who settled in Geneva after being appointed as Professor there, expressed interest for the collection. However, he could not purchase the findings for the museum of Geneva without a previous inspection. The worn-down and unprepared fossils were thus packed and sent to Geneva. However, after opening the crates, in them ‘not one piece seemed to be useful anymore’ and ‘annoyed letters were sent from Geneva to Genoa and from there further to Argentina’ (Weigelt, 1951). This situation led Roth to travel to Europe and, when necessary, put all the broken pieces back together. Roth was at the time 30 years, had four children, and was returning to his homeland 14 years after his emigration.

The encounter between Roth and Vogt is telling about their different fields of expertise between a fossil collector and in the far ground of the Pampa an academic zoologist. Gertrud Weigelt, a Swiss migrant in La Plata who wrote a detailed obituary about Roth likely from firsthand oral transmission, insightfully described the clash between Roth and Vogt. When Roth traveled to Switzerland to repair part of the collection that was damaged on the trip, Vogt encouraged him to take courses in comparative anatomy, geology and paleontology. Roth, who up to that point was entirely self-taught, was introduced to more methodical work that, indeed, improved the quality of his collecting methods. He also learned the technique of microscopy and the use of photography for scientific research. However, an episode in Geneva recorded by Weigelt reveals the valuable knowledge that Roth had acquired on the ground and the limits of working solely in universities in Europe. According to her, Roth gained the trust of Carl Vogt through a small incident.

- ‘One day, while mounting the foot of a *Panochtus* in the practical exercises, Vogt stopped by to check on the work of the students. As he approached the table, he asked: “What are you doing? This is wrong!”. Roth answered: “Professor, if this is wrong, you don’t know what the foot of a *Panochtus* looks like”. Annoyed, Vogt left the room. All attendants believed that Roth had messed up with “the Great Vogt”; but the opposite was the case. The next day, Vogt came to him, telling him he checked his work again and found it correct. Roth should continue his preparation. From then on, Roth became his favorite student; Vogt allowed him to work at his side and gave him private lessons next to the ordinary lectures.’

Carl Vogt, with whom Roth had these long contacts, was an influential zoologist, elected as a member to the American Philosophical Society in 1869. He is well-documented to have expressed racist and extreme views, as in the English version of one of his books, ‘Lectures on Man’ (Vogt, 1864). In it, Vogt wrote on how each human ‘race’ had evolved from a different type of ape and how the ‘white race’ was a separate species. It is unrecorded, as far as we know, if and how these views may have influenced Roth, or Roth’s opinion on them.

While Roth profited from the academic environment in Geneva, his main aim was selling collections, which was the sustain of his family in Argentina. It was not until 1880 that he got to sell a new collection to the University of Geneva. However, the financial possibilities there did not fulfill his expectations. In letters to his wife Elisabeth Schütz, he expressed his frustration (Weigelt, 1951):

- ‘A shame that you and the children are not here, otherwise I would repair the whole collection myself. If I would not need to hurry with selling so much, there is nothing else I could earn more money with … Sadly, Geneva does not have the money and only offered us 12'000 Francs. I wanted to sell it to them for 20'000. Professor Vogt has now written to Bern to see if both museums would be able to buy the collection together. If only the North Americans would come! Then I bet I could get 50'000 Francs for it. It is good I came here myself, because there is nobody else here who could repair such fossils.’

Using different criteria (minimal wage, inflation), a crude estimate of how much money 12′000 Swiss Francs in 1880 would represent today is provided by multiplying by 200 or 300, resulting in 2.4 or 3.6 million. Selling fossils was a profitable business (Carlini et al., 2016). Another example is based on a list of estimated prices from the archives at the Muséum d'histoire naturelle de Genève by the established fossil preparator and museum technician A. Dreyer in 1893 (Lang et al., 1892; Additional file 2). A ‘Toxodon’ is estimated at 10′000 Swiss Francs of the time, and the carapace and tail of a glyptodont at 4′500. Details of costs of Catalogue 5 fossils sold by Roth in Zurich are provided by Voglino et al. (2023).

Seven months after his first trip to Switzerland, Roth returned to Argentina in November 1880. Each way of the journey took 1 month. His partial success was twofold, as he, in addition to selling the collection, expanded his knowledge and learned much through his studies at the University of Geneva. From that time on, he was determined to devote the rest of his life to scientific research. When Roth arrived back in Argentina at a time of crisis because of bad harvests and land speculations that undermined the countries’ wealth, collecting ‘professionally’ now also served a newer, higher purpose. Roth and his family did not return to Pergamino, but instead settled in San Nicolas, declaring his special area of research from the large basin of Paraná, with its palaeontologically interesting steep banks, to the area of Entre Rios, first described by d’Orbigny and Darwin (Simpson, 1984). The fossil beds were well-known to the local people who used the fossil mollusks, especially the oysters, to make lime, and Roth probably was aware or knew the work of Bravard (1858) published in Paraná (Entre Ríos Province). It is important to remark that after D’Orbigny around 1827 (Tonni & Pasquali, 2006), Roth was one of the first who collected fossil vertebrates in these deposits.

An example of the importance given to the collection of fossils and sometimes its sales profit is offered by the words of the legislator José Hernández, well-known by his authorship of ‘Martín Fierro’, in a legislative session in the Buenos Aires provincial senate in December 1883. He emphasized the need for the Museo Público de Buenos Aires (the one in which Burmeister worked) to recruit a person to collect fossils, because the fossil mammals found in the Pampean region were sold and taken to Europe, and as an example he noted that in San Nicolás, a mister ‘Santiago Rut’ (Santiago Roth) had collected numerous fossils that have been sent to Copenhaguen (Bond, 1999).

Evidently, collecting practices were the ground where Roth built up his career. Although he provided important fossils to several leading paleontologists in a globalizing period of this discipline, Roth’s publications came much later. Arguably, a reason could have been his limited writing skills at the time. As Weigelt (1951) explained based on family letters and diverse documents, Roth’s ‘writing was not his forte, and it is questionable if the many publications would have come to be without his luck in finding a wife that had a clear mind and a brilliant education.’ Indeed, Elisabeth Schütz from Ranflüh obtained her teachers’ certification in 1871 at the erstwhile teachers’ seminar Hindelbank and was voted into the school ‘nach Aeschi bei Spiez’ (Kanton Bern). She went to Argentina in 1872 with her family and soon after found Santiago Roth, to whom she would be wife and loyal work associate thereafter. The extent of Elizabeth´s input on his husband’s publications should be further considered.

## Career milestones and scientific contributions: Roth’s discovery of the human from Fontezuela o Pontimelo

**Fig. 3:**
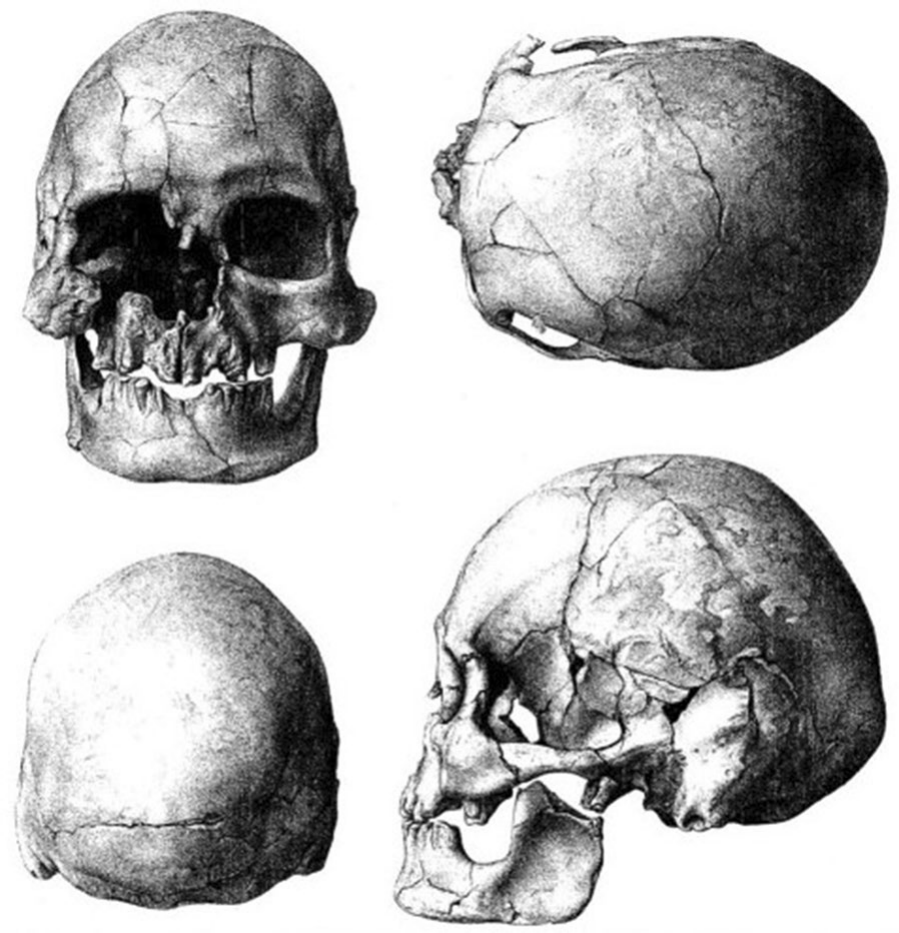
Skull of the Fontezuelas or Pontimelo skeleton as illustrated in Hansen (1888) and reproduced by Politis and Bonomo (2011, Fig. 4). The skull is illustrated with a photograph by Hansen (2019, p. 73, Fig. 42)

Roth found in 1881 a human skeleton underneath a glyptodont carapace in the vicinity of the Río Arrecifes, provincia de Buenos Aires. The place name Fontezuelas corresponds to the stream tributary of the Arrecifes River that is noted as ‘Pontezuelo’ or ‘Pontimelo’ in some maps and works. As with the Saladero skeleton, Roth’s discovery in Fontezuelas constitutes one of the first reports of the coexistence of man with the extinct fauna of the South American Quaternary. It was first depicted in Roth’s Catalogue number 2 (1884), and from 1889b there is an extensive letter of Roth to professor J. Kollmann on the discovery’s history. This finding, first described by Hansen (1888; Fig. 3), and that of another human skeleton by Baradero (Martin, 1907), also in Buenos Aires Province, convinced Roth (1888) of the concurrency of humans with the mammals of the Pampas formation, a view shared by others (Ameghino, 1889; Hansen, 1888; Lehmann-Nitsche, 1907; Virchow, 1883).

Carl Vogt (1881), based on information provided by Roth, pointed out that the human remains from ‘Fontezuelas’ were disarticulated and partially on surface in ‘pampeano superior’ sediments. Florentino Ameghino (1915:230) positioned the human of Fontezuelas in the upper Pliocene, wrote on some aspects of its anatomy, and taxonomically placed it in a new species, *Homo pliocenicus*. The contemporaneity of the human remains with those of the glyptodont were subject of debate. Roth and Ameghino supported it, whereas the North American Aleš Hrdlička (1869–1943) on the contrary attributed the association to a more recent intentional human burial (Politis & Bonomo, 2011). Bumeister (1884) saw the specimen, or at least part of it, but was not particularly impressed, stating that the lower jaw ‘seemed to me to show nothing deviating from the type of the native race’.

The skeleton of Fontezuelas was first deposited in Geneva but sent to the Museum of Zoology of the University of Copenhagen at the end of the nineteenth century, where it is still deposited with the accession number NHMD ZMK 11/1885 (Politis & Bonomo, 2011). The date of registering into the collection is July 10, 1885, shown in a showcase as ‘*Homo sapiens*, Pontimelo, Argentina’.

Politis and Bonomo (2011) reported on the dating of a phalanx of the skeleton kept in Copenhagen that provided an age of 1823–1927 cal yr BP. The carapace of *Glyptodon* lacked the necessary collagen for the standard dating method. The current view is that the human skeleton and the glyptodont carapace were not synchronous (Menéndez et al., 2015).

The ‘human of Fontezuela’ is one of many examples on how field discoveries by Roth triggered numerous discussions and exchanges by experts that ultimately addressed quite fundamental questions of paleontology, in this case involving the human species (Menéndez et al., 2023).

## Roth’s Patagonian expeditions and the Museo de La Plata

**Fig. 4:**
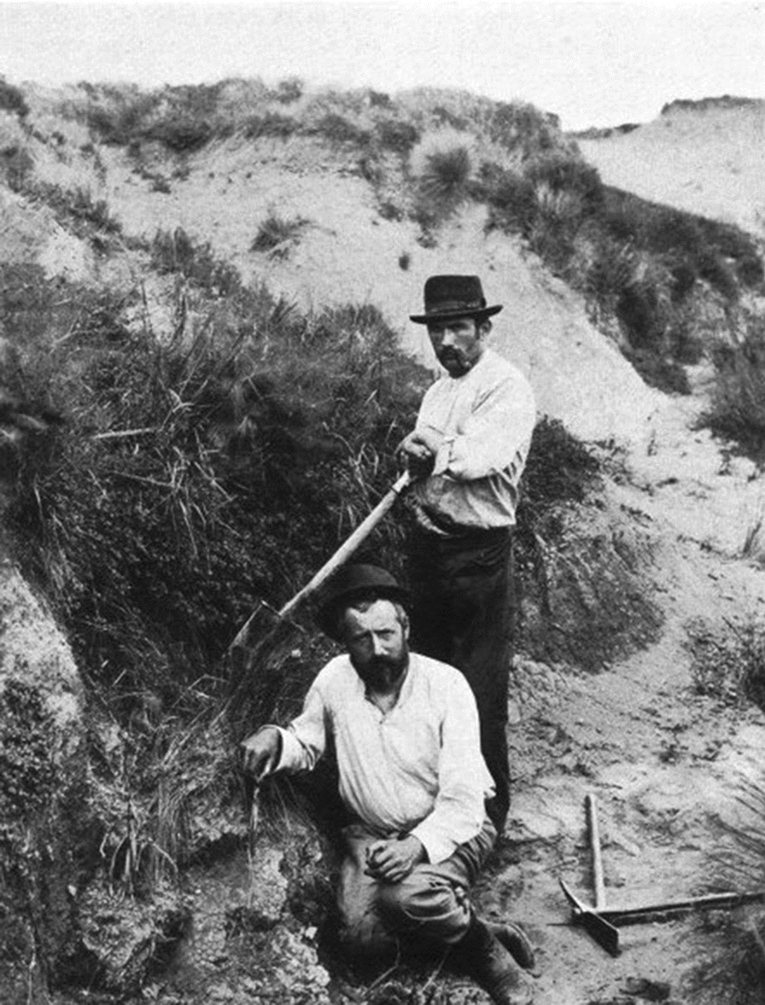
Santiago Roth (in a seated posture), presumably in the vicinity of Pergamino, Buenos Aires Province (Weigelt, 1951). Some sources indicate that the person standing might be François Machon, but this is unlikely, as is the report that this picture was taken during explorations in Patagonia of 1892. The photo may instead depict Roth working on a carapace of *Glyptodon* aided by a laborer in Pergamino in 1890 (Weigelt, 1951)

In 1892, the Swiss physician based on Argentina François Machon (b. 1862) carried out an expedition to the Argentinean Patagonia, and invited Santiago Roth to join (Sánchez del Olmo, 2021), one of many travels of Roth to Southern Argentina (Fig. 4). The expedition’s purpose was to look for lands to be colonized on a large scale, partially sponsored by the Barón Maurice de Hirsch of the Jewish Colonization Association. The departure of Machon and Roth was from Buenos Aires on March 4, 1892. They left Patagonia to Buenos Aires on July 9 of the same year by ship. They visited different localities of Neuquén, Río Negro and Chubut (Machon, 1893). At the time those areas were inhabited by indigenous and mestizo people, including, for example, ‘araucanos’ as indicated by Machon. Machon wrote a travel diary, took photographs, and collected numerous objects and human remains that, years later, he donated to different museums, i.e., Neuchatel (Sánchez del Olmo, 2017; 2021). In an obituary note on Roth, Machon (1925) noted Roth’s affability and good disposition towards him in this expedition. The latter and others led by Roth should be critically revisited given the dispossession of indigenous lands in Northern Patagonia, since the military campaign called ‘Conquista del Desierto’ between 1878 and 1885, led by the then War Minister General and later President Julio Argentino Roca (Larson, 2020). Even if Roth and Machon were not directly involved with the military advance given the time and mode of their expedition, they served for the purposes of territorial cognizance and scientific modernization. Both were tied to racist discourses about indigenous people in the formation of Argentinian state (see Sánchez del Olmo, 2021).

Weigelt (1951) reported that this experience in Patagonia was seen as a success, but upon returning Roth had financial difficulties to attend to. Roth’s brother-in-law from Genoa, the founder of the company Carlo F. Hofer & Co., allowed him credits in large forbearance repeatedly. Roth had to ask his family back in Switzerland for financial help. During these most difficult times for his family, they found accommodation in the house of Roth’s sisters in Rosario. The death of his father-in-law opened up the opportunity for them to temporarily move to his settlement in Baradero, but this was only a short-term solution based on financial circumstances.

**Fig. 5:**
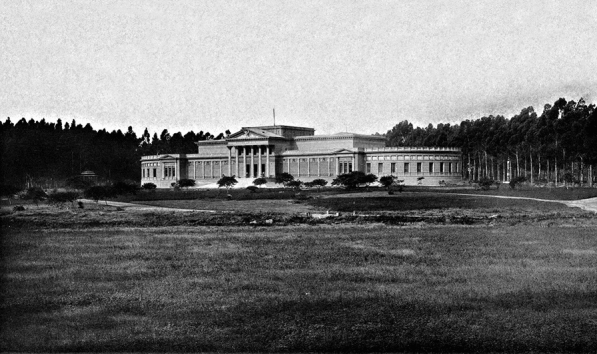
Building of the Museo de La Plata in 1900 (from Archivo Fotográfico Ministerio de Infraestructura)

In 1895 Francisco Pascasio Moreno (1852–1919), a prominent explorer and academic scientist in Argentina and the first director of the Museo de La Plata (Fig. 5), appointed Roth as Head of the Paleontological Section of the Museo de La Plata (*Jefe de la Sección Paleontología*), probably impressed by his experience as fossil collector and also the excellent observations made in his trip to Patagonia with Machon. This was also one of the main reasons Roth was appointed to make observations on the Andean region at the time of border issues with Chile (Machon, 1925). After a fleeting passage of Florentino Ameghino, the position now occupied by Roth at the Museum had been filled by the Swiss geologist Alcides Mercerat (Carrasquero, 2016; Farro, 2008; Podgorny, 2009; Simpson, 1984). Roth’s large family moved to La Plata. The catalogue of fossil mammals of the museum was started in 1898.

**Fig. 6:**
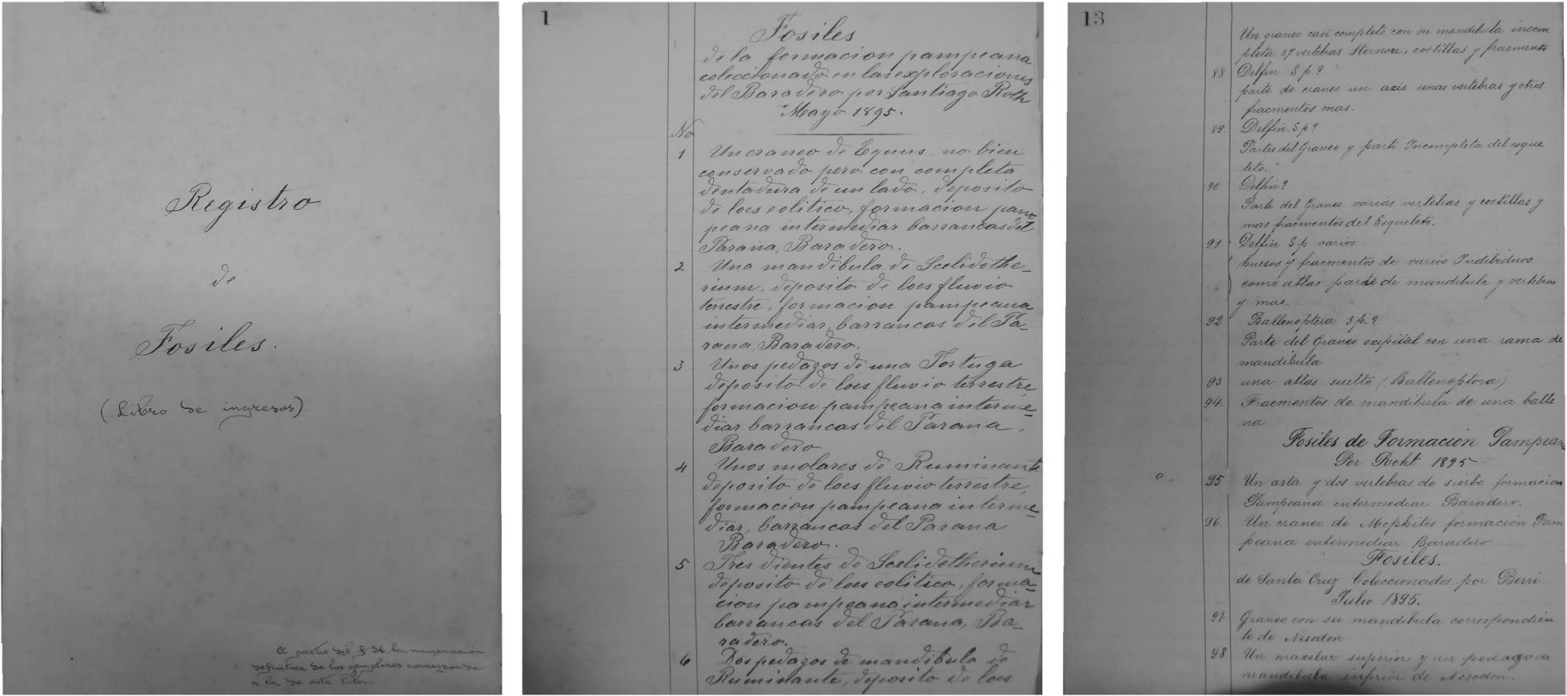
First Book Entry of the Sección Paleontología Vertebrados of the Museo de La Plata (‘Libro de Ingresos), 1895. First and second pages with the record of a Roth collection from Baradero, Buenos Aires Province (from Archives of the División Paleontología Vertebrados, Museo de La Plata)

In 1895 a fossil vertebrate collection collected by Roth in May of that year was registered but not catalogued in the Palaeontology Section of the Museo de La Plata (MLP Entrance Book of fossils—N° 1; Fig. 6). The collection consists of 183 specimens of fossil mammals, most of them coming from ‘Depósito de loes eolítico, formación pampeana intermedia Barranca del Paraná, Baradero’ and was probably sold by Roth to the Museo de La Plata.

Roth continued his work uninterruptedly at the Museum until 1924, the year of his death. From the incorporation of the Museum into the National University of La Plata Roth was Section Head and then Director of the School of Geological Sciences (1906–1907) and Head of the Geology Section (1907–1913) according to the changing section’s names. In 1919 the Palaeontology Section became independent again and was later separated into two sections, Roth was the Head of the Vertebrate Palaeontology Section until his death.

The Museo de La Plata was a key institution in the development of an identity of science and nation in Argentina (Andermann, 2007; Barquez & Díaz, 2014). The work of Santiago Roth in the Museo de La Plata was influential, although not necessarily reflected by the volume of his published work. First, the exploration commission of which he was a member fulfilled an arduous reconnaissance task, which was a subject of highest praise from Moreno.

**Fig. 7:**
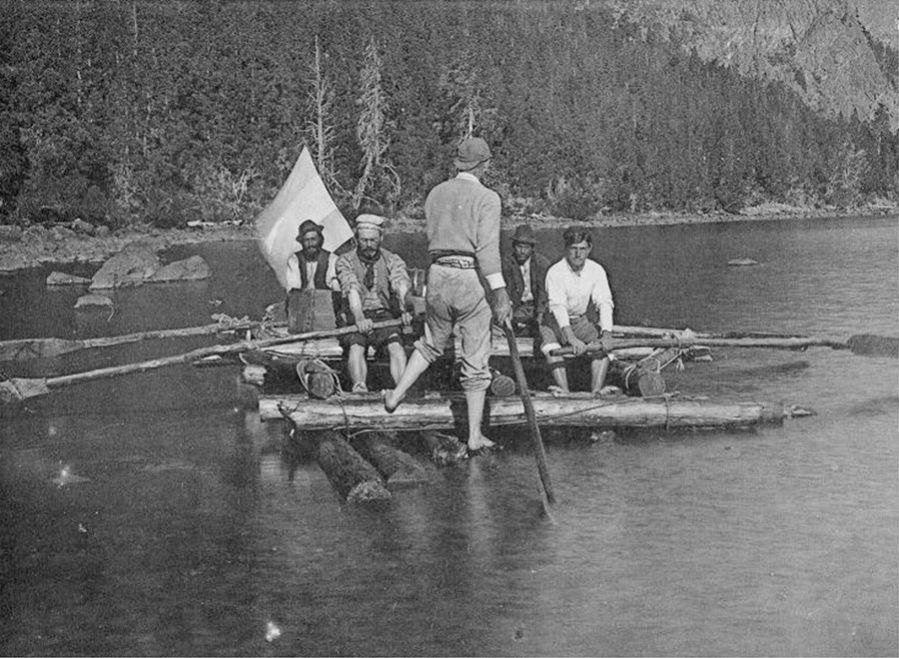
Staff of the Museo de La Plata rafting the Traful lake in 1896 (Moreno, 1898). Roth may be depicted in this picture, together with topographers that were part of the exploration team

**Fig. 8:**
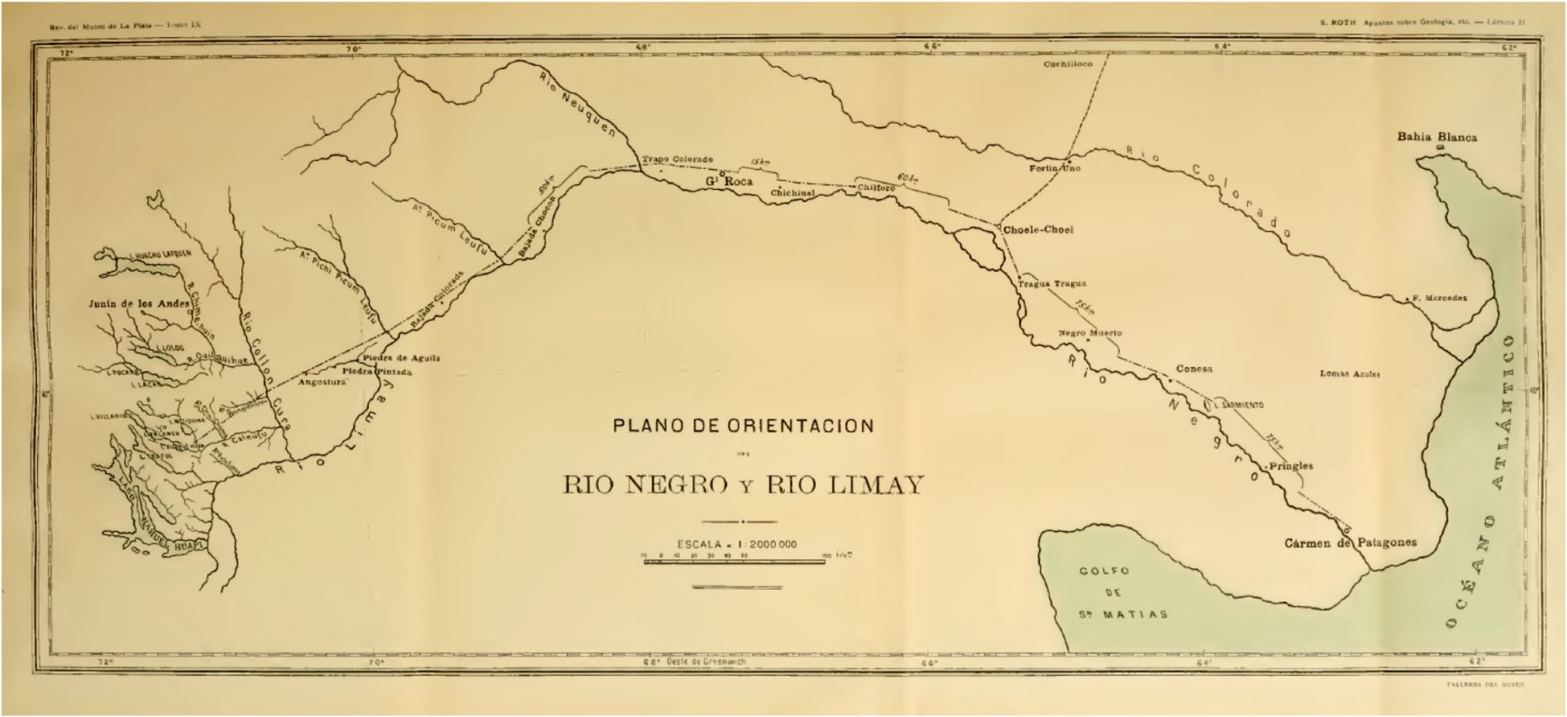
Map of the región in Northwestern Patagonia Areas explored by Roth from Roth’s (1899) ‘Apuntes sobre la geología y la paleontología de los territorios del Río Negro y Neuquén' (plate 2, ‘Plano de orientación del Rio Negro y Rio Limay’)

**Fig. 9:**
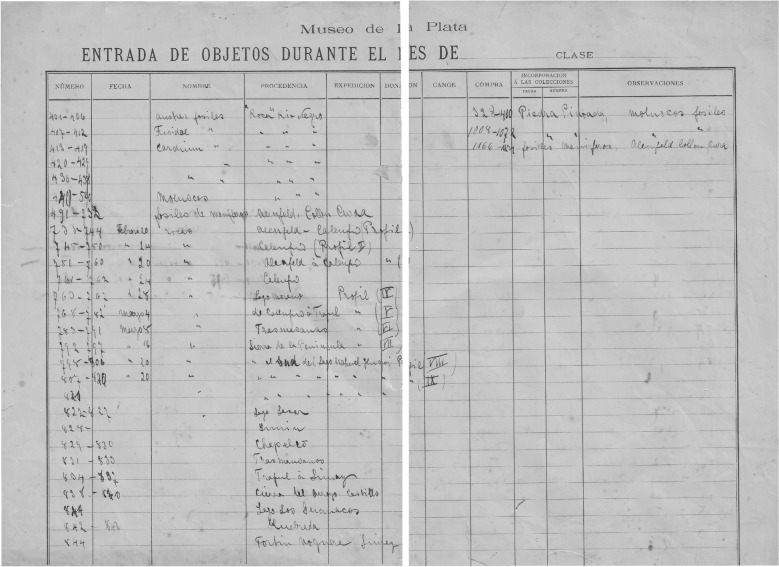
Museo de La Plata field inventory of geological and paleontological samples (*Museo de La Plata – Entrada de Objetos durante el mes de …*) handwritten by Santiago Roth during the trip to Río Negro and Neuquén territories in January–June of 1896. From Archives of the División Paleontología Vertebrados, Museo de La Plata

By 1895/1896 F.P. Moreno had a mandate from the Government of Argentina to work out a solution for the border disputes with Chile in the Andes region. Moreno commissioned Roth, as a geologist and paleontologist, two topographers, Adolfo Schiörbeck and Eimar Soot, and the assistant Juan M. Bernichan to make a trip to Río Negro, Limay, Collon-Curá rivers and Nahuel Huapi Lake (Fig. 7). In this campaign Roth sampled rocks and fossils in several localities and measured profiles. In February, 20, 1896 this commission reached Collón Curá rivers, Neuquén Province (Fig. 8). Santiago Roth collected approximately 240 fossil mammals in this area (lot 491–732, see Fig. 9). The gray tuffaceous sandstone beds outcropping along the inferior valley of the Collón Curá River were first mentioned by Roth (1899:156) and were formally described as the Collón Curá Formation by Yrigoyen (1969). The fossil mammals recovered there by Roth became the first basis for the recognition of the Colloncuran fauna (middle Miocene). The fossils were labeled by Roth with the initials ‘*T.m.C*.’ and yellow tag with sequential numbering. Later in 1899 Roth described and figured some of them, i.e., *Icochilus andiadys*.

One of the first tasks that Roth had to face in the Museo de La Plata was to adopt a cataloguing system for the fossil samples. In 1897 Roth used the same approach for all the fossils collected during a trip to Chubut, registering with initials the geographic precedence and numbering the samples in tags of different colours. The cataloguing system of fossil vertebrate collections changed in 1901 at the Museo de La Plata to another numerical system, i.e., 12-Notoungulata (Reguero, 1998).

**Fig. 10:**
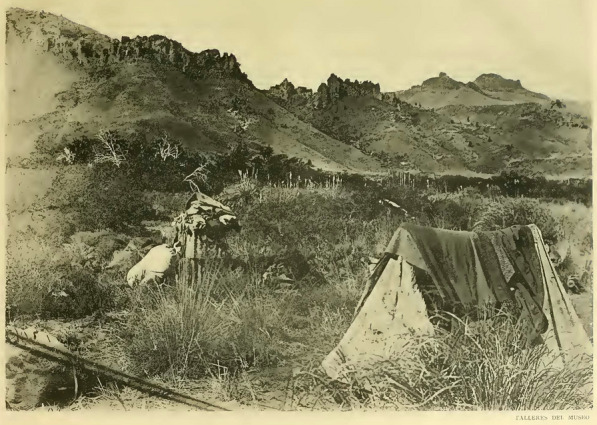
Photograph from an expedition by Roth in Neuquén province, in which exposures of Cretaceous rocks are shown. From Roth, 1899, plate 4, ‘Formación de tobas cretácicas atravesadas lor lava en la región del Río Caleufu’

Like the Ameghinos, Roth (1904) reported that the mammals from several of his Patagonian sites were contemporaries with dinosaurs, a confusion caused among other things by the incipient knowledge of Patagonian geology. However, Roth did find undoubtedly Cretaceous sites (Fig. 10), from which he extracted remains of dinosaurs and other reptiles. In an influential synthesis of the dinosaur finds in Argentina written by the eminent German paleontologist Friedrich von Huene (1929), many of the discoveries are attributed to Roth. Notes in the catalogue of dinosaurios of 1922 at the Departamento de Paleontología de Vertebrados of the Museo de La Plata include remains of dinosaurs found in 1897 in Neuquén. In addition, Roth found other dinosur remains in Neuquén and Río Negro during the Roth–Schiller–Gaggero expedition of the Museo de La Plata in January of 1922. Walter Schiller was a reknown geologist of German origin. Some of the reptiles found by Roth were described by the prominent paleontologist A. Smith Woodward, from the British Museum of Natural History, in London (Woodward, 1896). He described the Cretaceous crocodylomorphs: *Notosuchus terrestris* and *Cynodontosuchus rothi,* and later (Woodward, 1901) other discoveries by Roth in Cretaceous rocks, among them the first theropod dinosaur from South America, *Genyodectes serus* (Rauhut, 2004).

Several expeditions by Roth lasted 6–7 months. In addition to the geological and hydrological observations, Roth and his colleagues collected not only fossils, but also extant fauna and flora of Argentina as well as anthropological and ethnographical material of the regions, for all departments of the museum at the time. However, it is the paleontological collections which made the museum famous worldwide.

For the last expedition necessary for the border settlement between Chile and Argentina in 1902, different groups were on their way: Rudolf Hauthal (late director of the Roman Museum in Hildesheim) in Ultima Esperanza, Moreno with the English (acting as neutral experts) under the leadership of Colonel Holdich in the Andes, and Roth as third, who traveled with a caravan of 45 packed and just as many unpacked mules through Patagonia to the Nahuel Haupi Lake in thirteen major stages, to meet up there with Moreno and the English commissioners. Various wild watercourses had to be passed. Roth, who had known the area, found that things had changed. The rivers could sometimes no longer be crossed at the same spots as before. In a gorge called Angostura, where there was hardly any water previously, a wide river had now filled the whole basin. Where they used to make camp and find plenty of food for their horses, now was a trench hollowed out by water. Thus, Roth, who had ridden ahead for reconnaissance, had almost thought he would have to spend the night in the gorge with his companion, before he finally found a way out. Further details and anecdotes of the vicissitudes of this and other expeditions can be found in Weigelt (1951). One of them concerns the failed attempt by Roth to buy land in Patagonia to settle with his family there.

## Roth and the Pampean Formation—fieldwork and stratigraphic work in other regions of Argentina

Roth’s collections were made in sediments of different antiquities. The ones he carried out in Quaternary sediments of the Pampean Region were especially significant, because during his excavations, he developed a biostratigraphic and biochronological scheme that was advanced for his time. The scheme has been subject to multiple revisions and interpretations, important to understand the recent evolution of faunas in that region (e.g., Carrillo & Püschel, 2023; Carrillo-Briceño et al., 2023; Christen et al., 2023; Kerber, 2023; Le Verger, 2023; Ruiz-Ramoni et al., 2023; Voglino et al., 2023). In his collections Roth located each of the remains he found in his stratigraphic scheme, thus allowing the formation of faunal aggregates on which the prevailing past environmental conditions were interpreted.

In 1888 Roth published his first publication, a work on the Pampean Formation and its origin ‘*Über Entstehung und Alter der Pampasformation in Argentinien*’ (‘On the formation and age of the Pampas formation in Argentina’) in the *Zeitschrift der Deutschen Geologischen Gesellschaft*. The genesis of Pampas loess was a subject of intense debate, and Roth set up a new hypothesis, competing with works of Darwin, d’Orbigny, Bravard, Burmeister and Ameghino. A supporter of the mainly aeolian origin of such deposits, Roth thought that the Pampas loess was basically paleosol sequences (Imbellone & Teruggi, 1993). The stratigraphic work of Roth in the Argentinian Pampas was highly recognized until the time of Roth’s death (Kraglievich, 1925). Taken from his last work on the Pampean Formation (1921), his early views got more certainty through his later research (Voglino et al., 2023).

## Coming back: Roth’s visits to Switzerland and the Honorary Doctorate from the University of Zurich

In December of 1887 Roth traveled back to Switzerland again for a longer sojourn. He did so with his pregnant wife and seven children, the eighth child was born then in Zurich. Roth also brought along several parrots, wild cats, and other animals on this long journey (Weigelt, 1951). The returnees resided in the still existing Hotel Limmathof upon arrival, located only a few hundred meters away from the central building of the University of Zurich and the Swiss Polytechnic Zurich (ETH).

**Fig. 11:**
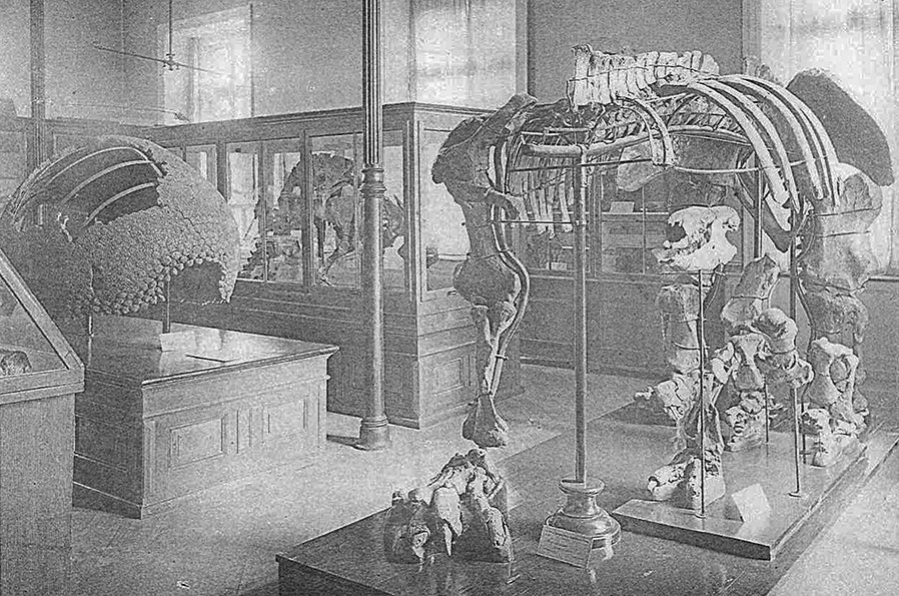
Photograph of mounted fossils from the collection Roth, exposed in the Swiss Polytechnic Zurich, ETH, 1893–1909

Settled in Zurich, while dealing with matters of military service and taxes (Weigelt, 1951), the nearly 40-year-old Roth visited the lectures of Albert Heim (1849–1937), the most famous Swiss geologist at the time, and joined geological excursions in the Alps with great interest, as well as Heim on his surveys of subterranean waterflows. He was also on the lookout for buyers of his collections. Two catalogues were published in Zurich; the one from 1889 comprised 284 numbers and concerns the collection still in Zurich (catalogue 5; Voglino et al., 2023), and the second one, No. 6 in his series of catalogues, comprising 136 numbers, was published in 1892 after his departure, under the care of his wife. It was not easy to sell the collections for what Roth considered they were worth. The correspondence at the time went to all large museums from Rome to Stockholm. The collection in catalogue No. 5 was acquired for Zurich with the help of state and canton (Schulthess, 1920; Voglino et al., 2023; Fig. 11). It was exhibited in a large glass display, becoming a highlight of the Zoological Museum (Voglino et al., 2023). Roth himself never saw this exhibition, because in 1891, as the purchase of fossils became possible, he was already back in Argentina and never returned to Switzerland.

**Fig. 12:**
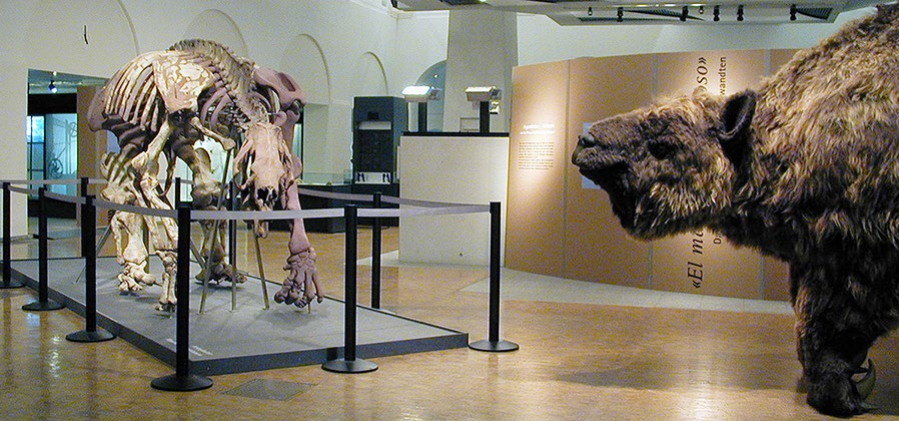
Hall at the entrance of the Zoological/Paleontological Museum of the University of Zurich as of 2000, with a skeleton and a reconstruction of a ground sloth *Megatherium americanum* collected by Santiago Roth, part of the temporary exhibit ‘El Mamífero Misterioso’ (Claude, 2000). Photo by Jürg Stauffer, courtesy of the Zoological Museum of the University of Zurich

In 2000, the Zoological Museum of the University of Zurich opened a temporary exhibit entitled ‘El Mamífero Misterioso’ (Claude, 2000), devoted to ground sloths and other fossils collected by Roth (Fig. 12). The reconstruction of the giant sloth at scale made for the exhibit sadly no longer exists, as the object was discarded by the Zoology Museum in 2018 on account of an infested coberture, the need to restore it, and the uncertainty whether the sloth had a skin as restored so far or rather as that of an elephant (I. Klussman, pers. comm. April 2023). Currently, instead, a local Triassic dinosaur is displayed there.

Before Roth returned to Argentina, he traveled regions with certain geological similarities to Argentina: the classic basins of Paris as well as the typical loess areas in Alsace. Thereupon he visited several museums of Europe and the geological and zoological institutes to personally get in touch with their directors. The result was rather disappointing. London and Paris would have only taken a few selected specimens. He was not very impressed by the presentation of his findings in Copenhagen, and he could not come to a deal with the museum in Berlin, with which he was in long negotiations with. However, the fifth collection could stay in Switzerland.

**Fig. 13:**
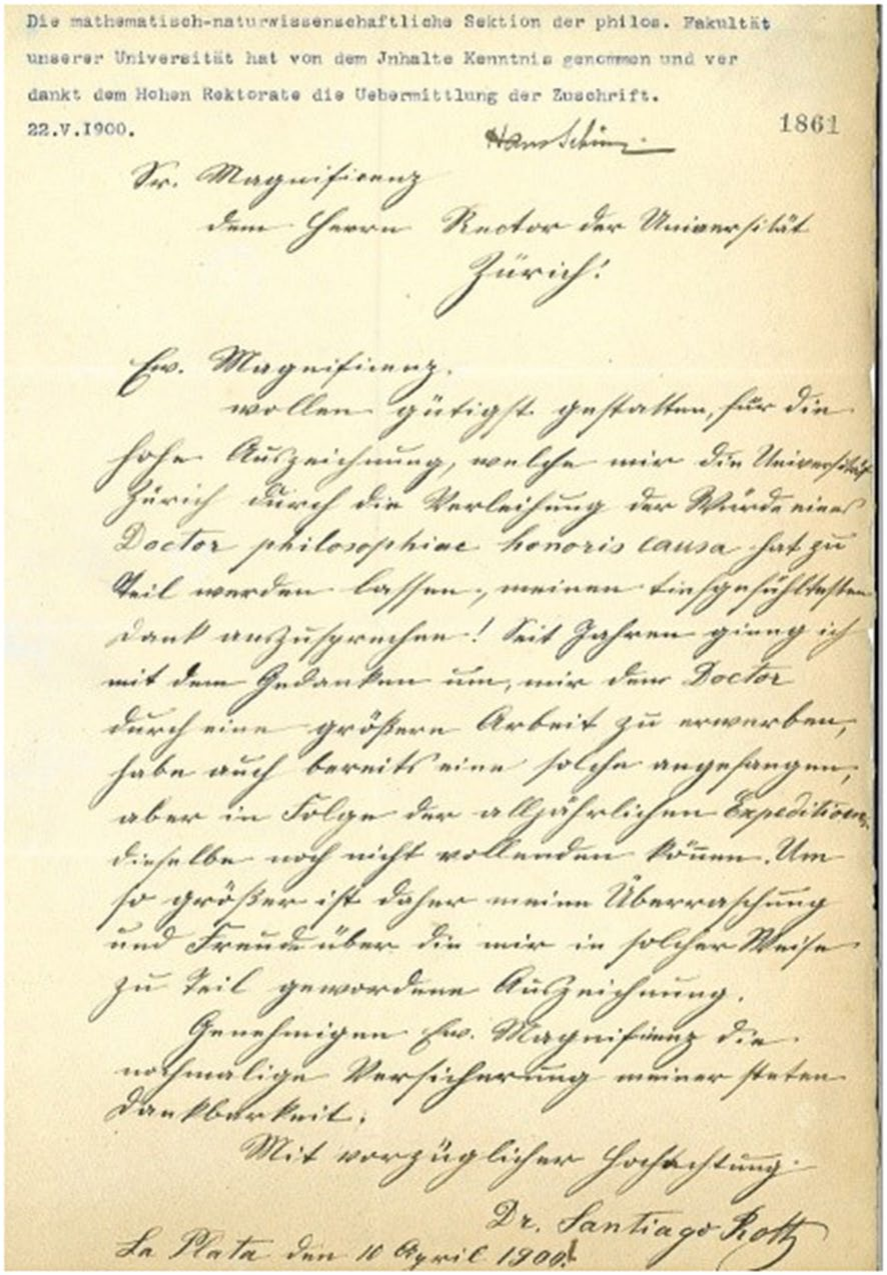
Copy of the letter by Santiago Roth to the President of the University of Zurich thanking him for the honorary title. An English translation is presented in the main text. See Additional file 4 for a German transcription

In 1900 the University of Zurich awarded Roth the honorary doctorate, Doctor Philosophiae Honoris Causa, ‘In Anerkennung seiner grossen Verdienste um die palaeontologische Erforschung von Südamerika und in dankbarer Erinnerung ansein, den Sammlungen seines Heimatlandes bewiesenes Interesse’ (Additional file 3), which can be translated as ‘In recognition of his great contribution to the paleontological study of South America and in grateful remembrance of his interest in the collections of his native country’. A letter by Santiago Roth to the President of the University of Zurich thanking for the honorary title has been preserved (Fig. 13). The text of the latter is transcribed (Additional file 4) and translated from German as follows:

- ‘To His Magnificence
- The Director of the University of Zurich!
- Your Magnificence
- in your kindness will allow me to express my deeply felt gratitude for the great distinction that the University of Zurich has bestowed on me by awarding me with the title/laureateship of a Doctor philosophiae honoris causa! For years I have been thinking about acquiring the Doctor title through a major study, which I had already started, but due to the recurring yearly expeditions I have not been able to complete said work. All the greater is my astonishment and joy for receiving this honor in such a manner.
- Allow me, Your Magnificence, to reiterate my continual gratefulness.
- With the highest regard
- Dr. Santiago Roth
- La Plata the 10th of April 1900’

Around that same time the ‘International Committee of Geological Correlation’ based on New York appointed him as a member, a commission which only eight scholars were part of, and he was the only representative of South America. W. D. Matthew, the illustrious paleontologist at the American Museum of Natural History in New York, said on the occasion of this appointment: ‘The valuable work of Dr. Santiago Roth carries the hallmark of an extensive scientist, that is as much experienced as sensible, is fully familiar with the disputed formations through continuous observation and is also an expert of the Tertiary fauna of Europe, that he used for his comparisons’ (In Weigelt, 1951).

## Roth hydrological works

In 1908 Roth accepted a new task: the direction of the Geological–Topographical Institute of the Buenos Aires Province. In this context, he had to lead the making of hydrographic maps and investigations for the acquisition of drinking water. During this time, he continued his position in the Sección Paleontología de Vertebrados of the Museo de La Plata *ad honorem.* Roth tried to make use of the deep drillings for scientific work from the start. In the course of 9 years, he supervised over 100 drillings, some of which went down to 1000 m. The geological results were presented in a summary work (Roth, 1921). The hydrological studies opened up many undeveloped and barren areas to livestock breeding and agriculture by discovering the subterranean watercourses, while other areas that did not meet the necessary hydrographic conditions for colonization were abandoned.

Roth traveled to the provinces of Cordoba, Tucuman, Santiago del Estero, Catamarca, and Salta in 1907/1908, commissioned by the parliament. He also made a private stop up in Jujuy, where the train went up to a height of 4000 m above the ocean level at the time. On the journeys for these special missions Roth would receive a rail carriage with a sleeping room, living room and kitchen including a private cook. Roth had lived over half a year in the rail carriage during the supervision of the drillings in Santiago del Estero Province. He was accompanied by a geology student as an adjutant. The carriage could be attached to or detached from a train at any time and also reach the private railroads of the sawmills. Therefore, once at the doorstep of a resident who had invited Roth, he could enter the house directly from the carriage. Other than on rail lines, some forest areas were hardly passable at all. Water also had to be transported there by train, so logging families from far and wide would flock to the tanker trains.

Roth found the long-sought drinking water-table after half a year of intense labor in Sancho Corral and Añatuya for the entire Chaco line. The problem was not easy to solve, due to the water being salty not only at great depths but also at the surface and, therefore, only the middle layers, that were sealed by non-porous layers, would yield useable water.

Weigelt (1951) recounted on the communication of Roth with his family on the challenges of fieldwork and his celebrated accomplishment helping people’s lives as a result of his hydrological explorations. On his travels Roth got infected with malaria, from which he suffered to the end of his life. In a letter of March 30 of 1908 (Additional file 5), Roth informed the Director of the Museum of La Plata, Samuel Lafone Quevedo (1835–1920), that he was recovering from malaria (‘paludismo’) in Tucumán. In the letter he also mentioned the studies carried on the sources for the provision of drinking water for Tucumán, making some recommendations for the place to build a dam on the river Salí.

Around 1909 Roth was engaged in large canal construction projects for irrigation as well as means of transport in the Rio Negro region intended for the ‘civilatory development’ of those areas. Due to their temperate climate Roth tried, without success, to mediate on behalf of his countrymen from Switzerland for emigration and settlement, especially because the land would have been free for that purpose.

## 50 years since the arrival in Argentina and Roth’s death

Roth received a significant honor in 1916, when the Universidad de La Plata organized a public anniversary celebration in commemoration of the 50th year after his arrival in Argentina and handed over a commemorative coin as award for his services to the country and for 20 years of supervision of the paleontological collection. At his thank-you speech Roth mentioned in a humorous way that on the morning he set foot on the country, he also made his first geological discovery (Weigelt, 1951). At the time, the new arrivals had to be brought from the ship to the landing stage with ox teams when the water level was low, and during that he noticed that the wet ground was firm yet looked like clay. The boy was so interested, he had to go to the beach during luggage inspection and observe the strange rock from close up. ‘Therefore’, Roth concluded,

- ‘It has been truly fifty years today since I engaged in the problem of the Pampas formation! In this land, that has become so bountiful for science and has presented us with so many surprises, discoveries have been made that have shaped our understanding of earth’s history. In this land Darwin got the idea that the organic world, the same as everything else on earth, is subjected to constant evolution. In this land I have fought the speculative theories that were not in accordance with observation, and here I will continue to fight, to let the truth break through. If I’m not around anymore, the collected specimens will serve further studies. My heart will forever be connected with this earth.’

After this honour, Roth lived eight more busy years. At some point he underwent surgery when cataracts began to hinder him. Roth put much value in ensuring that the paleontological collection remained in the best organization and conservation state possible, to be accessed by specialists for further work.

In 1921/22, in his 70 s, Roth went on another journey through Patagonia to Chile (see above on the Roth–Schiller–Gaggero expedition) and published those results together with the ones from the official publications of 1897–99. Another work on mammalian dentitions of large scope entitled ‘La diferenciación del sistema dentario en los Ungulados, Notoungulados y Primates’ was published in 1927, 3 years after his death.

Roth (1927) discussed the evolution of the crown morphology and cusps of the teeth of many fossil mammals of Patagonia, mainly discovered and named by him, and how this related to major theories developed at the time on the homologies of mammalian molar cusps in general. Roth strongly supported the tritubercular theory, especially the ideas of Osborn, still valid today, being thus opposed to ideas of Ameghino on this subject. Further work revised the age of the fossils and the identity of some of the cusps as presented by Roth, but fundamental ideas have been tested and confirmed. In Roth’s (1927) work specimens, many of the taxa he described but had not figured previously, were illustrated.

Roth (1927) was part of a more extensive work planned by Roth, but which his eye cataracts difficulted. Roth presumably remembered the words of his former master Carl Vogt, that one must write assuming that the reader may have no previous knowledge on the subject (Fernández, 1925). Considering that there were no works in Spanish of the tritubercular dental theory, Roth made an introduction of that subject (mainly translating works in German), then discussing the differentiation of different tooth types.

Roth’s (1927) posthumous work was edited by Dr. Miguel Fernández, a well-known zoologist who was Roth’s friend. M. Fernández (1882–1950) was of Argentine father and German mother, studied in Zurich with Alfredo Lang and obtained his Doctorate in 1904. Looking for a candidate to occupy the post of professorship in Zoology, Roth wrote to A. Lang in Zurich, who suggested the name of Fernández. In 1906, by invitation of S. Lafone Quevedo, Director of the Museum of La Plata, M. Fernández became professor of Zoology and chief of its Department, beginning a friendship with S. Roth.

In excursions between 1922 and 1923, accompanied by the preparators Bernardo Eugui and Octavio Fernández, Roth verified observations taken on previous trips (1897–1899), correcting some annotations from his work on geological investigations in northern Patagonia (Roth, 1924).

During the 1922 expedition, Roth discovered the skeleton of a sauropod dinosaur that later became described as *Titanosaurus australis* by Richard Lydekker (1893), renamed *Neuquensaurus australis* (Salgado et al., 2005), whose assembly in the rooms of the Museo de La Plata was initiated by Roth himself and completed under the direction of his successor, Ángel Cabrera (1875–1960) (Giacchino & Gurovich, 2001; Torres, 1930).

**Fig. 14:**
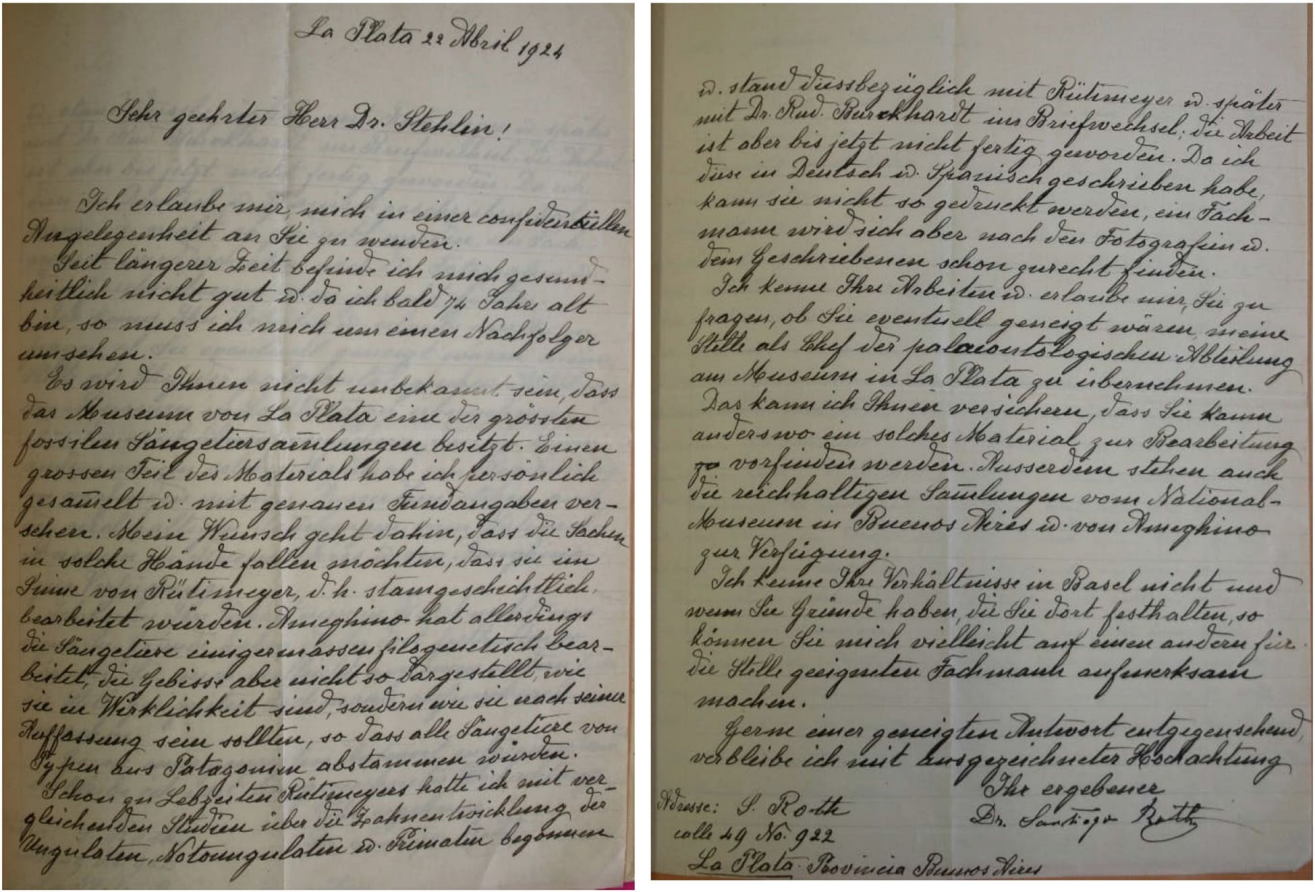
Letter of Roth inviting the paleontologist Hans Georg Stehlin from Basel to become his successor at the Museo de La Plata. Courtesy of Dr. Loïc Costeur, from archives at the Naturhistorisches Museum in Basel

Roth tried to bring a colleague from Switzerland as his successor at the Museo de La Plata. He contacted Hans Georg Stehlin in Basel (1870–1941) (Fig. 14; Additional file 6), as well as Paul Arbenz (1880–1943) in Bern, but both were bound to long-term occupations in Switzerland. The letter to Stehlin is transcribed and translated from German as follows:

- ‘Dear Doctor Stehlin!
- I take the liberty of contacting you with a confidential matter. For a while now I have not been in good health and since I am soon to be 74 years of age, I must search for a successor
- It must not have escaped your attention that the Museum of La Plata owns one of the largest fossil mammal collections. A large part of the material I have collected myself and have labelled it with an exact description of its provenance. My wish is that these items fall into the hands of someone who will analyze them in accordance with Rütimeyer, i.e., in a phylogenetic way. Ameghino has admittedly examined the mammals to a degree phylogenetically; however, he did not describe the dentition accurately, rather as he thought they ought to be, so that they fit the view that all mammals would have been derived from types from Patagonia.
- Already during his Rütimeyer’s lifetime I started with comparative studies on the tooth development of ungulates, Notoungulata and primates and corresponding with Rütimeyer and thereafter with Dr. Rud. Burckhardt on the matter. However, to this day the study remains unfinished. Since I wrote it in German and Spanish, it cannot be printed this way, but an expert will be able to work with the provided pictures and text.
- I know your work and take the liberty to ask you if you might be inclined to take on my position as the head of the paleontological department of the Museum La Plata. I can guarantee that it will be very hard to find a place with comparable material to work with. In addition, you would have access to the comprehensive collections of the National Museum of Buenos Aires and of Ameghino.
- I don’t know your circumstances in Basel and if you have reasons that keep you there, maybe you could point me towards a suitable expert for this position.
- I am hopeful for a positive answer, and remain respectfully yours
- Yours sincerely
- Dr. Santiago Roth
- Address: S. Roth
- calle 49 No. 922
- La Plata Provincia Buenos Aires’
- Towards the end of 1923, Roth participated in the festivities of his golden marriage anniversary in a circle of 20 grandchildren, but he was already terminally ill. Treated by his wife and eldest daughter with much dedication, he lived through his last months in the house of his eldest son in Buenos Aires, where he died on the night from the 3rd to the 4th of August 1924 (Weigelt, 1951). In the presence of a large circle of friends and official delegations of the scientific institutes from La Plata and Buenos Aires, Roth was buried in the German cemetery of Buenos Aires (Scheinsohn, 2021). The Swiss flag covered the coffin.

## Santiago Roth’s scientific legacy and his influence

All the obituaries of Roth by people who knew him personally praised his generous and amicable disposition. It is reported that Roth hosted many visitors at his home in an informal family setting, and how supportive he was of other visitors and immigrants from Europe (Machon, 1925; Weigelt, 1951). As a friendly professor and colleague, Roth contributed to forge a scientific community in the field of paleontology on a local and international level. However, his influence went far beyond his contemporaries.

The emergence of paleontology in Argentina was a field with much debates and personalities involved. Particularly, Roth was a contemporary of the most influential pioneer of vertebrate paleontology in Argentina, as well as the rest of South America, Florentino Ameghino (1853–1911). As it happened in other regions of the world, including the infamous case of Cope and Marsh in North America towards the end of the nineteenth century (Romer, 1964), there were rivalries and conflicts among paleontologists working in Argentina. As a paleontologist at the Museo de La Plata and Moreno's collaborator, Santiago Roth was involved in the personal conflicts between Florentino Ameghino and Francisco Moreno resulting during formative years of the museums in Buenos Aires and La Plata (Fernícola, 2011). Through various publications, Roth and Ameghino expressed accusations and objections regarding the capacity of their opponent and the accuracy of their taxonomic determinations and conclusions about geology and fossil mammals (Scheinson, 2021). Simpson (1962) discussed how the etymology of some of the genera and species names erected by Roth and Ameghino may hide jokes or puns that refer to controversies between them. In her obituary of Roth, Weigelt (1951) emphasized that besides the rivalry between Florentino Ameghino and Santiago Roth, there were also numerous anecdotes of mutual praise and amicable interactions.

A competition was generated in the search and exploitation of fossil deposits between Roth and Carlos Ameghino, who collected fossils for his brother Florentino. Although this struggle was beneficial in terms of the discovery and description of numerous vertebrate fossils, especially mammals, it motivated the mutual concealment of the discovered sites, providing unclear references as to their location. Such is the case of a series of fruitful deposits in an area north of Lakes Colhue Huapi and Musters, whose names Roth inverted in his notes, perhaps as a way of outwitting Carlos Ameghino. The exact location of several fossil sites discovered by them is unknown (Richard Madden pers. commun. March 2023) and attempts have been made to solve this issue (e.g., Fernicola et al., 2014; Marshall, 1990).

The description of location of the outcrops of the fossils collected in Patagonia by Carlos Ameghino by Florentino Ameghino has little information of provenance, in many cases none, so later workers had to infer them (Simpson, 1967a). Roth collected many fossil mammals and reptiles in Patagonia; many of the mammal sites are a little better known than Ameghino’s ones. The collecting areas for the Miocene mammals of the Collón Cura sites are located in maps with more precise information in Roth’s publications; in some lists of collected material he provided the exact location were the fossils were found. Rocks were also collected, all with his corresponding collection number. The Paleogene mammals collected in Patagonia have some places well-located, as the fossil mammals of the Gaiman Region, Chubut, that came from the Cerro Pan de Azucar (Simpson, 1936). Roth collected also many mammals in what he thought to be Cretaceous beds, that now we know are Eoecene in age, of what is called the Mustersan South American Land Mammal Age (SALMA). Many of these fossils Roth were marked with the letters ‘C.s.M.’, meaning ‘Cretáceo Superior del lago Musters’, after the expeditions and latter collectings, it is almost certain that this collecting place of Roth is the oriental slope of Cerro del Humo, north of Lake Colhue Huapi (Simpson, 1936). One of the fossils collected by Carlos Ameghino and described by Florentino Ameghino is the type of the astrapotherian *Astraponotus assymmetrus*, now in the Museo Argentino de Ciencias Naturales of Buenos Aires, which incidentally gave the original name to the ‘Astraponoteen Étage’ now the Mustersan SALMA and was collected in a locality called by Ameghino Colhuapi Norte. Some specimens in the Roth Collection in the Museum of La Plata of *Astraponotus assymmetrus* belong to the same individual as some collected by Carlos Ameghino; the Roth specimens come from the ‘C.s.M’ locality at Cerro del Humo, proving that Cerro del Humo and Colhuapi Norte are the same place, and Carlos Ameghino went in fact to some of the places in which Roth had collected specimens (Bond & Deschamps, 2010). Other places of Roth, such as Cañadón Blanco, have not been exactly located but Roth made a map of Patagonia, originally in the Sección Paleontología Vertebrados of the Museo de La Plata, in which he marked with some accuracy many of those localities. This map is lost, but fortunately Simpson made a copy and many years latter a copy of this map was sent to the Sección (Bond, unpublished).

Roth’s lasting influence was surely his discovery of sites and fossils and occasionally writings or collaborations on diverse issues concerning the biostratigraphy of the southern cone of the continent and its relation to other areas of the world, and taxonomy, biogeography, and extinction of diverse mammals and other vertebrates. His stratigraphic work influenced also the ideas of workers on invertebrate fossils of the time, with whom he maintained correspondence (von Ihering, 1895).

Roth was one of the first authors to recognize that the endemic native ungulates of the Cenozoic and Quaternary had originated and evolved in South America from forms immigrated from North America, as had been suggested by previous authors in this field, such as Gaudry and Scott (Simpson, 1980). In contrast, Florentino Ameghino, who described the largest number of species of these ungulates, considered that many of them were ancestral or corresponded to the different groups of ungulates in the northern hemisphere (Podgorny, 2005). Our current and robust understanding of this matter supports Roth’s view. In a work published in 1903, Roth substantiated his views and coined the term Notoungulata for one such native group, precisely the one whose representatives include the toxodont.

In his last monograph on the geology of the Pampean region, Roth included observations relevant to the event later named the Great American Biotic Interchange (Cione et al., 2015), pointing out a chronological sequence of immigrant taxa from the North and their contemporaneous endemic taxa, including carnivorans and other taxa (Ruiz-Ramoni et al., 2023).

Roth made discoveries of fossils in their stratigraphic context of great importance for later refinement of the land mammal ages in Patagonia. Particularly noteworthy are discoveries leading eventually to the recognition of the Colloncuran Land Mammal age (associated with the toxodont *Palyeidodon*) and the ‘Astraponoteen plus superieur’ (associated with the notohippid notoungulate *Eomorphippus*). These strata are both located chronologically in between classically recognized units, and the re-study of Roth’s sites and discoveries has led to major geochronological insights (Flynn et al., 2003; Madden et al., 2010). The recognition of the ‘Astraponoteen le plus superieur’ of Ameghino, originally a younger part of his Astraponoteen ëtage, latter the Mustersan SALMA, was the result of a study of fossils found by Roth in a site called ‘Cañadón Blanco’ of uncertain location in Chubut Province. In this site notoungulate taxa occurred (e.g., the notohippid *Eomorphippus obscurus*) that showed a fauna deemed intermediate between the Mustersan and the Deseadan SALMAs. These beds, some of them now recognized to outcrop also in the Gran Barranca in Chubut, are now referred to as the Tinguirican Salma, recognized posteriorly in Chile. Its age is referred to the Upper Eocene to Lower Oligocene (Flynn et al., 2003).

Roth named 111 species (Table 1). Similar to most other pioneer work based on unknown fauna, many of these species are invalid today (nearly 30% in this case), since more complete material or analysis of population variation have demonstrated they represent already described taxa. An additional large amount (35%) need taxonomic revision, since those have been evaluated as being later considered nomina vana, nuda or dubia.

**Table 1:** Species named by Santiago Roth

Sparassodonta
*Plesiofelis schlosseri* Roth, 1903b
*Plesiofelis cretaceus* Roth, 1903b (invalid species, junior synonym of *Plesiofelis schlosseri* Roth, 1903b
“Condylarthra”
Didolodontidae
*Xesmodon langi* (Roth, 1899a) (original Nomination *Glyphodon langi* Roth, 1899a)
?*Xesmodon proIixus* (Roth, 1899a) (*Megacrodon prolixus* Roth, 1899a) species inquirenda
*Megacrodon planus* Roth, 1899a (nomen dubium)
Macraucheniidae
*Polymorphis lechei* Roth, 1899a
*Polyacrodon ligatus* Roth, 1899a (invalid species, junior synonym of *Polymorphis lechei* Roth, 1899a
Proterotheriidae
*Anisolambda nodulosa* Roth, 1903b
*Anisolophus minusculus* (Roth, 1899a) (original nomination *Diadiaphorus minusculus* Roth, 1899a)
*Heteroglyphis dewoletzky* Roth, 1899a
Litopterna incertae sedis
*Polyacrodon lanciformis* Roth, 1899a
*Lambdaconus elegans* Roth, 1903b (lost holotype)
*Proacrodon transformatus* Roth, 1899a (lost holotype)
Nototungulata
Henricosborniidae
*Henricosbornia minuta* (Roth, 1903b) (original nomination *Monolophodon minutus* Roth, 1903b)
Notostylopidae
*Otronia muhlbergi* Roth, 1901a
*Orthogenium ameghinoi* Roth, 1901a (invalid species, junior synonym of *Notostylops murinus* Ameghino, 1897
Interatheriidae
*Icochilus andiadys* Roth, 1899a (treated as *Protypotherium endiadys* by Vera et al., 2017)
Mesotheriidae
*Nesciotherium indiculus* Roth, 1899a
*Eutrachytherus modestus* Roth, 1899a
*Eutypotherium lehmann-nitschei* Roth, 1901a
*Typotherium lausenii* Roth, 1894 (invalid species, junior synonym of *Mesotherium cristatum* Serrés, 1867)
Archaeohyracidae
*Archaeohyrax graciIis* Roth, 1903b (invalid species, junior synonym of *Eohegetotherium priscum* Ameghino, 1901)
*Archaeotypotherium transitum* Roth, 1903b (invalid species, junior synonym of *Archaeotypotherium propheticus* (Ameghino, 1897)
*Degonia kollmanni* Roth, 1901a (invalid species, junior synonym of *Pseudhyrax eutrachytheroides* Ameghino, 1901)
*Pseudopithecus modestus* Roth, 1901a (invalid species, junior synonym of *Pseudhyrax eutrachytheroides* Ameghino, 1901)
*RankeIia elegans* Roth, 1901a (invalid species, junior synonym of de *Pseudhyrax eutrachytheroides* Ameghino, 1901)
Hegetotheriidae
*Hegetotherium andinum* Roth, 1899a (invalid species, junior synonym of *Hegetotherium mirabile* Ameghino, 1887)
*Pachyrucos depressus* (Roth, 1899a) (original nomination *Propachyrucos depressus* Roth, 1899a) nomen dubium
*Pachyrucos medianus* (Roth, 1899a) (original nomination *Propachyrucos medianus* Roth, 1899a) nomen dubium
*Propachyrucos robustus* Roth, 1899a (?*Hemihegetotherium robustum* or nomen dubium)
Notohippidae
*Eurystomus stehlini* Roth, 1901a (invalid species, junior synonym of *Eomorphippus obscurus* Ameghino, 1901)
*Lonkus rugei* Roth, 1901a (invalid species, junior synonym of *Eomorphippus obscurus* Ameghino, 1901)
Notohippidae?
*Puelia plicata* Roth, 1901a
Isotemnidae
*Periphragnis palmeri* (Roth, 1903b) (original nomination *Calodontotherium palmeri* Roth, 1903b)
*Calodontotherium varietatum* Roth, 1903b (invalid species, junior synonym of *Periphragnis harmeri* Roth,1899a)
*Colhuapia rosei* Roth, 1901a (nomen dubium) (cf. *Periphagni*s sp.)
*Colhuelia frühi* Roth, 1901a (nomen dubium)
*Rhyphodon angusticephalus* (Roth, 1903b) (original nomination *Eurystephanodon angusticephalus* Roth, 1903b)
*Eurystephanodon cattanii* Roth, 1903b (invalid species, junior synonym of *Periphragnis harmeri* Roth,1899a)
*Eurystephanodon crassatus* Roth, 1903b (nomen dubium)
*Lafkenia schmidti* Roth, 1901a (nomen dubium)
*Lafkenia sulcifera* Roth, 1901a (nomen dubium) (cf. *Rhyphodon* sp.)
*Isotemnus haugi* (Roth, 1901a) (original nomination *Lelfunia haugi* Roth, 1901a)
*Lemudeus angustidens* Roth, 1903b (invalid species, junior synonym of *Periphragnis harmeri* Roth,1899a)
*Lemudeus proportionalis* Roth, 1903b (invalid species, junior synonym of *Rhyphodon angusticephalus* (Roth, 1903b))
*Pehuenia insigna* Roth, 1903b (invalid species, junior synonym of *Rhyphodon lankesteri* Roth, 1899a)
*Pehuenia magna* Roth, 1903b (nomen dubium)
*Pehuenia wehrlii* Roth, 1901a (invalid species, junior synonym of *Rhyphodon lankesteri* Roth, 1899a)
*Periphragnis cristatus* Roth, 1903b (invalid species, junior synonym of *Periphragnis harmeri* Roth, 1899a)
*Periphragnis harmeri* Roth, 1899a
*Rhyphodon lankesteri* Roth, 1899a
*Setebos terribiIis* Roth, 1901a (invalid species, junior synonym of *Rhyphodon lankesteri* Roth, 1899a
*Distylophorus alouatinus* (Roth, 1901a) (original nomination *Stylophorus alouatinus* Roth, 1901a)
*Tehuelia regia* Roth, 1901a (invalid species, junior synonym of *Periphragnis harmeri* Roth, 1899a)
*Thomashuxleya rankei* Roth, 1901a (invalid species, junior synonym of *Periphragnis harmeri* Roth, 1899a)
*Trigonolophodon modicus* Roth, 1903b (nomen dubium, cf. *Periphragnis* sp.)
Homalodotheriidae
*Trigonolophodon inflatus* Roth, 1903b
*Diplodonops ampliatus* (Roth, 1901a) (original nomination *Diplodon ampIiatus* Roth, 1901a) (nomen vanum)
Homalodotheriidae?
*Heterolophodon ampliatus* Roth, 1903b
Toxodontidae
*Haplodontherium darwini* Roth, 1888 (nomen dubium)
*Haplodontherium monlezuni* Roth, 1888 (nomen dubium)
*Nesodontopsis burckardti* Roth, 1899a (likely synonym of *Hyperoxotodon speciosus* (Ameghino, 1887))
*Nesodontopsis deformis* Roth, 1899a (likely synonym of *Hyperoxotodon speciosus* (Ameghino, 1887))
*Nesodontopsis speciosus* Roth, 1899a (invalid species, junior synonym of *Hyperoxotodon speciosus* (Ameghino, 1887))
*Hypotoxodon primigenius* (Roth, 1927) (original nomination *Palaeotoxodon primigenius* Roth, 1927) (nomen dubium)
*Palyeidodon obtusum* Roth, 1899
*Plesioxotodon tapalquensis* Roth, 1901a (nomen dubium)
*Toxodon elongatus* Roth, 1895a (invalid species, junior synonym of *Toxodon ensenadensis* Ameghino, 1889)
Notoungulata incertae sedis
*Degonia sympathica* Roth, 1901a (nomen dubium)
*Ortholophodon prolongus* Roth, 1901a (nomen dubium)
*Pyramidon klaatschi* Roth, 1901a (nomen dubium)
*Trigonolophodon elegans* Roth, 1903b (nomen dubium)
*Ultrapithecus robustus* Roth, 1901a (nomen dubium)
Astrapotheria
*Albertogaudrya robusta* Roth, 1903b
*Trigonostylops gegenbauri* (Roth, 1899a) (original nomination *Staurodon gegenbauri* Roth, 1899a)
*Chiodon supernus* (Roth, 1899a) (original nomination *Staurodon supernus* Roth, 1899a) (nomen dubium)
*Blastoconus robertsoni* Roth, 1903b (nomen dubium)
*Grypholophodon imperfectus* Roth, 1903b (nomen dubium)
*Grypholophodon morenoi* Roth, 1903b
*Grypholophodon tuberculosus* Roth, 1903b
*Helicolophodon giganteus* Roth, 1903b (invalid species, junior synonym of *Parastrapotherium holmbergi* Ameghino, 1895)
*Megalophodon dilatatus* Roth, 1903b (invalid species, junior synonym of *Astraponotus assymmetrus* Ameghino, 1901)
*Megalophodon thompsoni* Roth, 1903b (invalid species, junior synonym of *Astraponotus assymmetrus* Ameghino, 1901)
*Monoeidodon primus* Roth, 1899a
*Notamynus dicksoni* Roth, 1903b (invalid species, junior synonym of *Astraponotus assymmetrus* Ameghino, 1901)
*Notamynus holdichi* Roth, 1903b (invalid species, junior synonym of *Astraponotus assymmetrus* Ameghino, 1901)
*Notorhinus denticulatus* Roth, 1903b (nomen dubium)
*Tonorhinus haroldi* (Roth, 1993b) (original nomination *Notorhinus haroldi* Roth, 1903b) (nomen dubium)
*lsolophodon aplanatus* Roth, 1903b
*Isolophodon cingulosus* Roth, 1903b
Equiidae
*Hippidion saldiasi* (Roth, 1899b) (original nomination *Onohippidium saldiasi* Roth, 1899b)
Cervidae
*Coassus entrerianus* Roth, 1903b
Mammalia “Ungulata” incertae sedis
*Eutrochodon inceptus* Roth, 1903b
*lsolophodon aplanatus* Roth, 1903b
*Picunia nitida* Roth, 1901a (nomen dubium)
*Prostylophorus margeriei* Roth, 1901a (nomen dubium)
*Trilobodon brancoi* Roth, 1901a (nomen dubium)
Rodentia
*Megastus elongatus* Roth, 1899a
*Myopotamus fossilis* Roth fide Rusconi, 1929b (nomen nudum)
Glyptodontidae
*Propalaehoplophorus informis* Roth, 1899a
*Hoplophorus kelleri* Roth, 1888 (nomen nudum)
*Hoplophorus studeri* Roth, 1888 (nomen nudum)
*Panochthus beyrichi* Roth, 1888 (nomen nudum)
*Panochthus vogti* Roth, 1888 (nomen nudum)
*Glyptodon damesi* Roth, 1888 (nomen nudum)
*Plesiomegatherium hansmeyeri* Roth, 1911a
*Grypotherium domesticum* Roth, 1899b (invalid species, junior synonym of *Mylodon darwini*)
*Grypotherium moeschi* Roth, 1888 (nomen nudum)
*Grypotherium rutimeyeri* Roth, 1888 (nomen nudum)
*Lestodon vogti* Roth, 1888 (nomen nudum)
*Diellipsodon heimi* (Roth, 1899a) (original designation *Elipsodon heimi* Roth, 1899a)

Several works cited here as Roth 1901a, Simpson cited in 1948 also as 1901, but in Simpson (1967b), in the second part of the ‘Beginning’ work, Simpson stated how these works should be cited as from 1902, as that is the year in which they were presumably edited—as cited by A. S. Romer in his ‘Bibliography of fossil Vertebrates’. Concerning *Propachyrucos robustus* Roth, 1899a, Vera (2019) considered this taxon to be allocated to *Hemihegetotherium* cf *tantillum*, but Seoane and Cerdeño (2020) considered *Propachyrucos robustus* a nomen dubium, pointing out its great resemblance to *Hegetotherium mirabile* (see also Seoane et al., 2019)

Roth defined in 1903 the Notoungulata, a major extinct group of South American native ungulates. This work is particularly important as it has a detailed study of the posterior and auditory region of the Notoungulata showing that members of this group can be differentiated from other South American endemic ungulates and those of the northern hemisphere. Roth stated in this work that some ‘families’ (e.g., Notohippidae) that Ameghino assumed to be related to the Equidae, were undoubtedly notoungulates.

Several researchers from the time and later renowned paleontologists dedicated vertebrate species to Roth in recognition of his work. These include *Cynodontosuchus rothi* Woodward, 1896, *Polydolops rothi* Simpson, 1935*, Chasicotherium rothi* Cabrera and Kraguevich, 1931, *Adelphotherium rothi* Mercerat, 1891, *Prototrigodon rothi* Kraglievich 1930, and *Scalabrinitherium rothi* Ameghino, 1885. A more recent example is *Santiagorothia chiliensis* (Hitz et al., 2000).

As part of his tasks at the Museo de La Plata, Roth set up displays of South American fossil mammals. He supervised the assembly of skeletons in realistic life postures hypothesized for extinct animals (Giacchino & Gurovich, 2001). Roth obtained copies of different casts of dinosaurs and mammals from the American Museum of Natural History, probably as part of an exchange of materials for exhibition and study.

The lack of infrastructure and the logistical challenges in the largely unexplored and extensive areas of Southern Argentina in which Santiago Roth made so many discoveries and geological observations are notable (Riccardi, 2017). In this sense Roth was a pioneer and his expeditions have been deemed by critical experts in the field as not less epochal than the celebrated Gobi Desert and Mongolian expeditions of the American Museum of Natural History (Reig, 1962). The exploratory and subsequent descriptive work of Roth (and of the Ameghinos) led to other major expeditions in subsequent decades that set the agenda for studies of extinct faunas of the continent to this day. Notable examples of expeditions during Roth’s life include those of Universidad de Princeton by John Bell Hatcher (1861–1904) to Santa Cruz (1896–1899), André Tournouër and Albert Gaudry (1827–1908) commissioned by the Muséum National d’Histoire Naturelle in París, and the Patagonian expeditions by the US Americans Frederic B. Loomis between 1911 and 1912 (Simpson, 1984).

Here lies a major legacy of Roth: the exploration for fundamental, primary data, which together with the work of the Ameghino brothers (Simpson, 1984) surely provided a tradition of taxonomic work of great importance in Argentinian paleontology to this day. Without falling into some naïve discourse of heroism when describing Roth’s work—his motives were surely in part economical and his limitations as a scholar a matter that could be studied in greater depth than done here—one can admire the exploratory work and the discoveries made. Even more so these days in paleontology, with the growing importance of meta-analyses in detriment of primary descriptive work (Korn, 2014; Plotnick, 2007; Polly, 2014) that ultimately provides the data for such studies. Fieldwork and the study of the resulting fossils in their stratigraphic context are the core, fundamental activities to provide empirical bases for the understanding of biotas of deep time. Here lies Roth’s scientific legacy.

###  Supplementary Information

**Additional file 1.** Examples of fossil mammals collected by Santiago Roth, in the collections in Copenhagen and La Plata.

**Additional file 2.** Estimates of prices of Santiago Roth's fossils in Geneva by A. Dreyer.

**Additional file 3.** Title from the University of Zurich of Doctor Philosophiae Honoris Causa to Santiago Roth (1900).

**Additional file 4.** Transcription of the letter by Santiago Roth to the President of the University of Zurich thanking him for the honorary doctorate title.

**Additional file 5.** Letter of 1908 in which Santiago Roth informed the Director of the Museum de La Plata that he was recovering from malaria in Tucumán and about his hydrological works searching for drinkable water for the region.

**Additional file 6.** Transcription of the Letter from Santiago Roth to Hans Georg Stehlin in Basel inviting him to be his successor at the Museo de La Plata.

## Data Availability

Archives of the University of Zurich, archives of the Naturhistorisches Museum Basel, archives of the Muséum d’Histoire naturelle Geneva, and Archivos del Museo de La Plata.
