# Supplementary material for: From fossil trader to paleontologist: on Swiss-born naturalist Santiago Roth and his scientific contributions
Source: Swiss J Palaeontol. 2023 Sep 11;142(1):19. doi: 10.1186/s13358-023-00282-6 (PMC10495517; doi:10.1186/s13358-023-00282-6)
Supplement: Supplementary file 1 — Additional file 1. Examples of fossil mammals collected by Santiago Roth, in the collections in Copenhagen and La Plata. [file 13358_2023_282_MOESM1_ESM.docx]

**Additional information**

**From fossil trader to palaeontologist: On Swiss-born naturalist Santiago Roth and his scientific contributions**

Marcelo R. Sánchez-Villagra, Mariano Bond, Marcelo Reguero, Tomás Bartoletti

**Contents**

1. Examples of fossil mammals collected by Santiago Roth, in the collections in Copenhagen and La Plata.

2. Estimates prices of Roth's fossils in Geneva by A. Dreyer. Supplementary Information

3. Title from the University of Zurich of Doctor Philosophiae Honoris Causa to Santiago Roth (1900).

4. Transcription of the letter by Santiago Roth to the President of the University of Zurich thanking him for the honorary doctorate title.

5. Letter of 1908 in which Roth informed the Director of the Museum de La Plata that he was recovering from malaria in Tucumán and about his hydrological works searching for drinkable water for the region in question.

6. Transcription of the Letter from Santiago Roth to Hans Georg Stehlin in Basel inviting him to be his successor at the Museo de La Plata.

**Additional Information 1**. Examples of fossil mammals collected by Santiago Roth, in the collections in Copenhagen and La Plata.


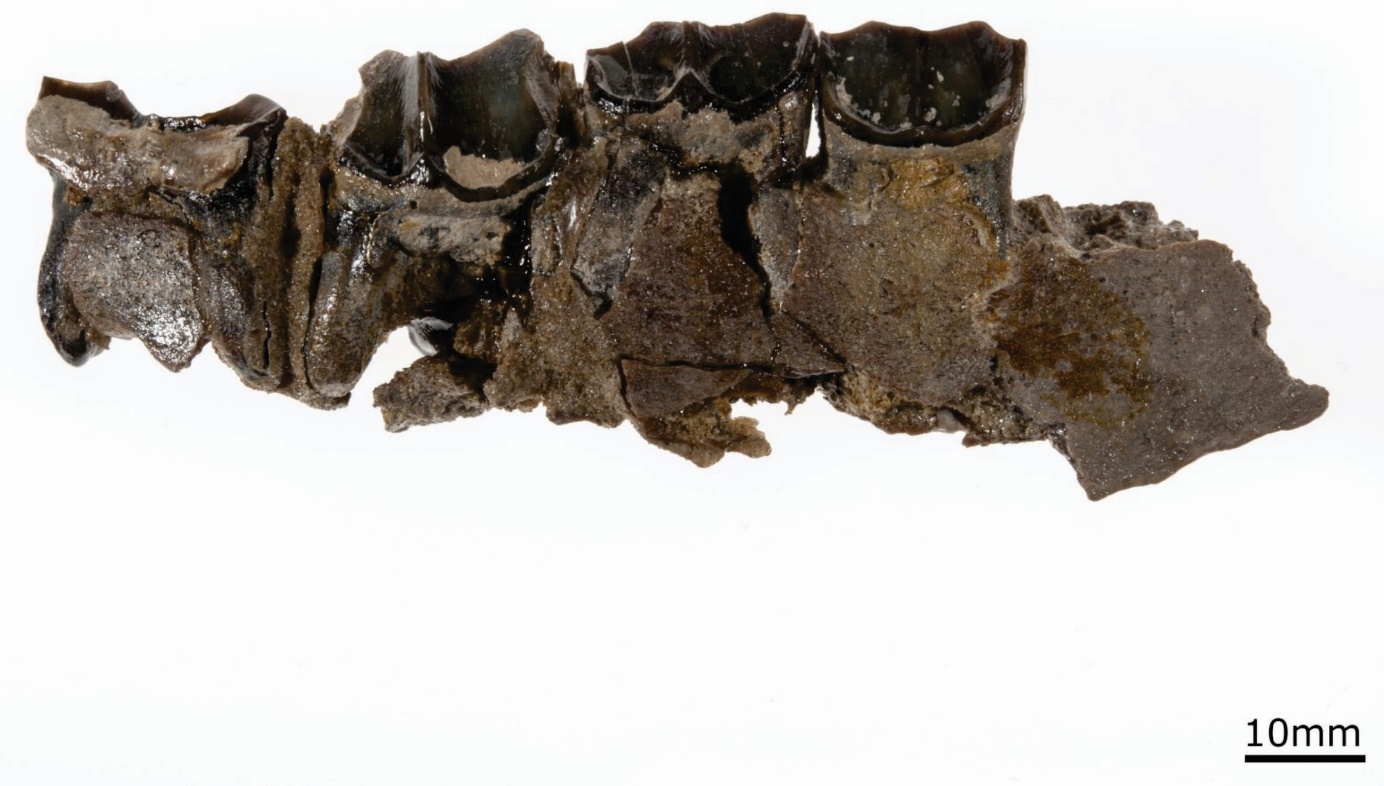


**Additional Information 1A.** Type of *Neoprocavia mesopotamica* Ameghino, 1889, Z.M.K. 111/1887, buccal view (Photo: Kasper Lykke Hansen, Hansen, 2020: Fig. 5)


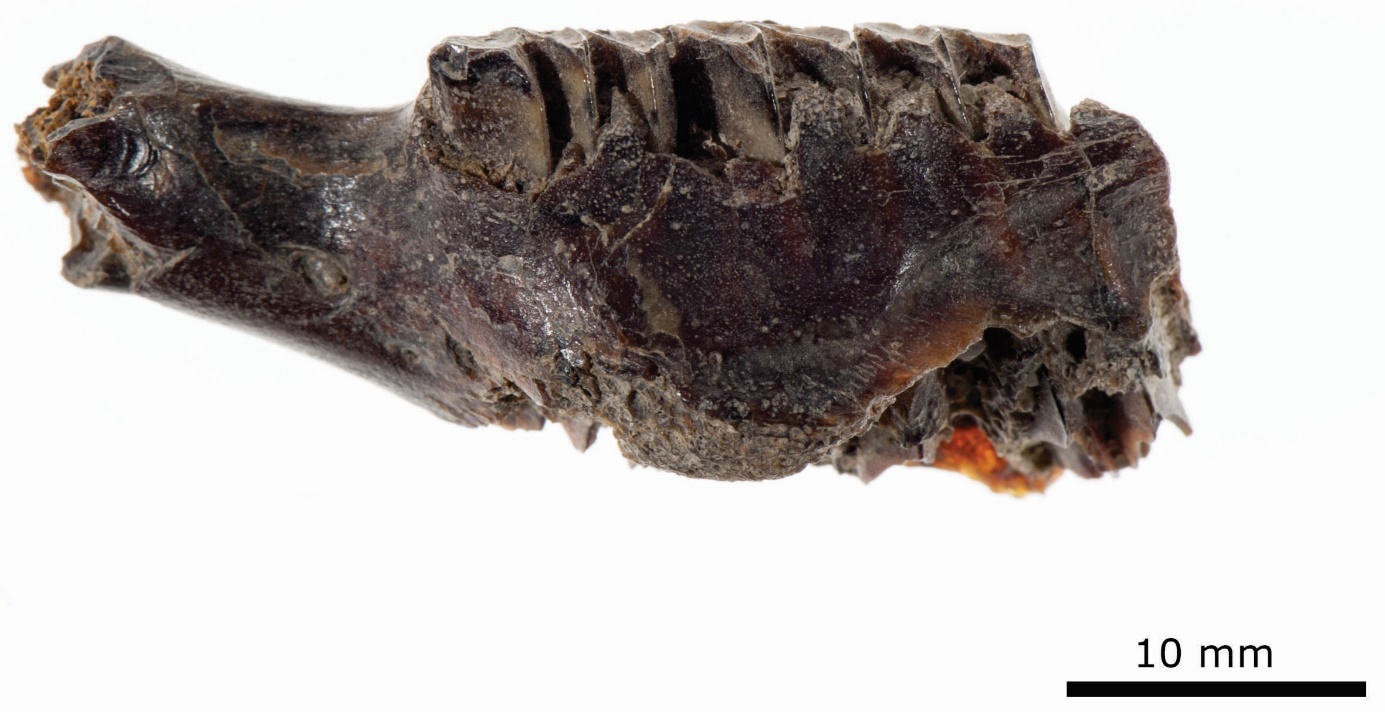


**Additional Information 1B.** Type of *Scalabrinitherium rothi* Ameghino, 1882, Z.M.K. 116/1887, buccal view (Photo: Kasper Lykke Hansen, Hansen 2020: Fig.4)

**
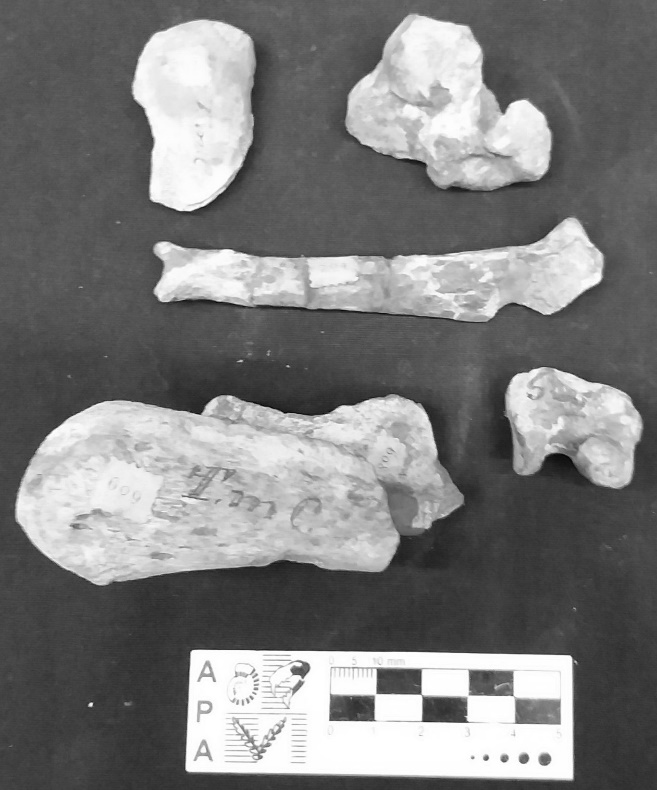
**

**Additional Information 1C.** Fossil mammals recovered by Roth in 1896 from the Collón Curá Formation at the Museo de La Plata.


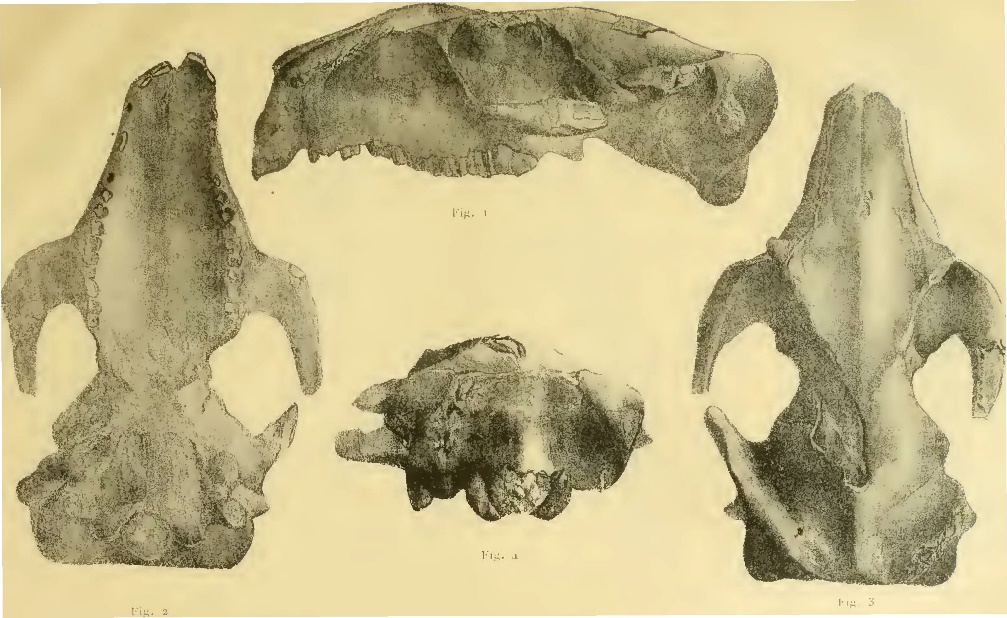


**Additional Information 1D.** *Icochilus andiadys*, skull (Roth, 1908, Revista del Museo de La Plata, Tomo XI, Lám. VI)

**
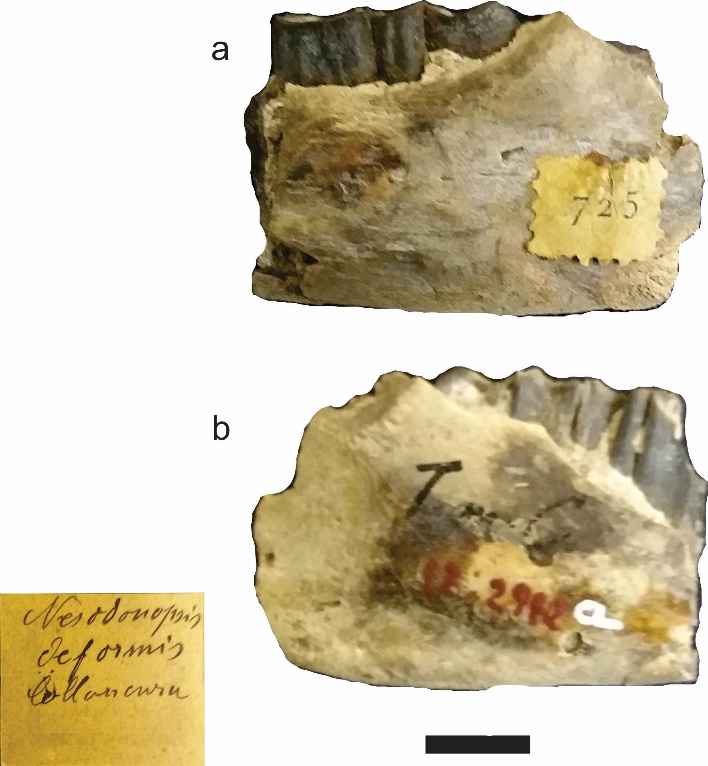
**

**Additional Information 1E.** Holotype of *Nesodonopsis deformis* Roth, 1903 from Collon Curá catalogued MLP 12-2912a, left mandibular fragment with m1-3. Labeled on the fossil “*T.m.C.*“ and yellow label 735.
