# Supplementary material for: From fossil trader to paleontologist: on Swiss-born naturalist Santiago Roth and his scientific contributions
Source: Swiss J Palaeontol. 2023 Sep 11;142(1):19. doi: 10.1186/s13358-023-00282-6 (PMC10495517; doi:10.1186/s13358-023-00282-6)
Supplement: Supplementary file 4 — Additional file 4. Transcription of the letter by Santiago Roth to the President of the University of Zurich thanking him for the honorary doctorate title. [file 13358_2023_282_MOESM4_ESM.docx]

**Additional information**

**From fossil trader to palaeontologist: On Swiss-born naturalist Santiago Roth and his scientific contributions**

Marcelo R. Sánchez-Villagra, Mariano Bond, Marcelo Reguero, Tomás Bartoletti

**Contents**

1. Examples of fossil mammals collected by Santiago Roth, in the collections in Copenhagen and La Plata.

2. Estimates prices of Roth's fossils in Geneva by A. Dreyer. Supplementary Information

3. Title from the University of Zurich of Doctor Philosophiae Honoris Causa to Santiago Roth (1900).

4. Transcription of the letter by Santiago Roth to the President of the University of Zurich thanking him for the honorary doctorate title.

5. Letter of 1908 in which Roth informed the Director of the Museum de La Plata that he was recovering from malaria in Tucumán and about his hydrological works searching for drinkable water for the region in question.

6. Transcription of the Letter from Santiago Roth to Hans Georg Stehlin in Basel inviting him to be his successor at the Museo de La Plata.

**Additional Information 4**. Transcription of the letter by Santiago Roth to the President of the University of Zurich thanking him for the honorary doctorate title.

“Sr. Magnifizenz

Dem Herren Rector der Universität Zürich!

Ew. Magnifizenz

wollen gütigst gestatten, für die hohe Auszeichnung, welche mir die Universität

Zürich durch die Verleihung der Würde eines Doctor philosophiae honoris causa hat zu Teil werden lassen, meinen tiefgefühlten Dank auszusprechen! Seit Jahren ging ich mit dem Gedanken um, mir den Doctor durch eine grössere Arbeit zu erwerben, habe auch bereits eine solche angefangen, aber in Folge der alljährlichen Expeditionen dieselbe noch nicht vollenden können. Um so grösser ist dafür meine Überraschung und Freude über die mir in solcher Weise zu Teil gewordenen Auszeichnung.

Genehmigen Eur. Magnifizenz die nochmaligen Versicherung meiner steten

Dankbarkeit.

Mit vorzüglicher Hochachtung

Dr. Santiago Roth

La Plata den 10 April 1900”
