# Supplementary material for: From fossil trader to paleontologist: on Swiss-born naturalist Santiago Roth and his scientific contributions
Source: Swiss J Palaeontol. 2023 Sep 11;142(1):19. doi: 10.1186/s13358-023-00282-6 (PMC10495517; doi:10.1186/s13358-023-00282-6)

**Additional information**

**From fossil trader to palaeontologist: On Swiss-born naturalist Santiago Roth and his scientific contributions**

Marcelo R. Sánchez-Villagra, Mariano Bond, Marcelo Reguero, Tomás Bartoletti

**Contents**

1. Examples of fossil mammals collected by Santiago Roth, in the collections in Copenhagen and La Plata.

2. Estimates prices of Roth's fossils in Geneva by A. Dreyer. Supplementary Information

3. Title from the University of Zurich of Doctor Philosophiae Honoris Causa to Santiago Roth (1900).

4. Transcription of the letter by Santiago Roth to the President of the University of Zurich thanking him for the honorary doctorate title.

5. Letter of 1908 in which Roth informed the Director of the Museum de La Plata that he was recovering from malaria in Tucumán and about his hydrological works searching for drinkable water for the region in question.

6. Transcription of the Letter from Santiago Roth to Hans Georg Stehlin in Basel inviting him to be his successor at the Museo de La Plata.

**Additional Information 5.** Letter of March 30 of 1908 (first and last page of 3) in which Roth informed the Director of the Museum de La Plata that he was recovering from malaria in Tucumán and about his hydrological works searching for drinkable water for the region in question.


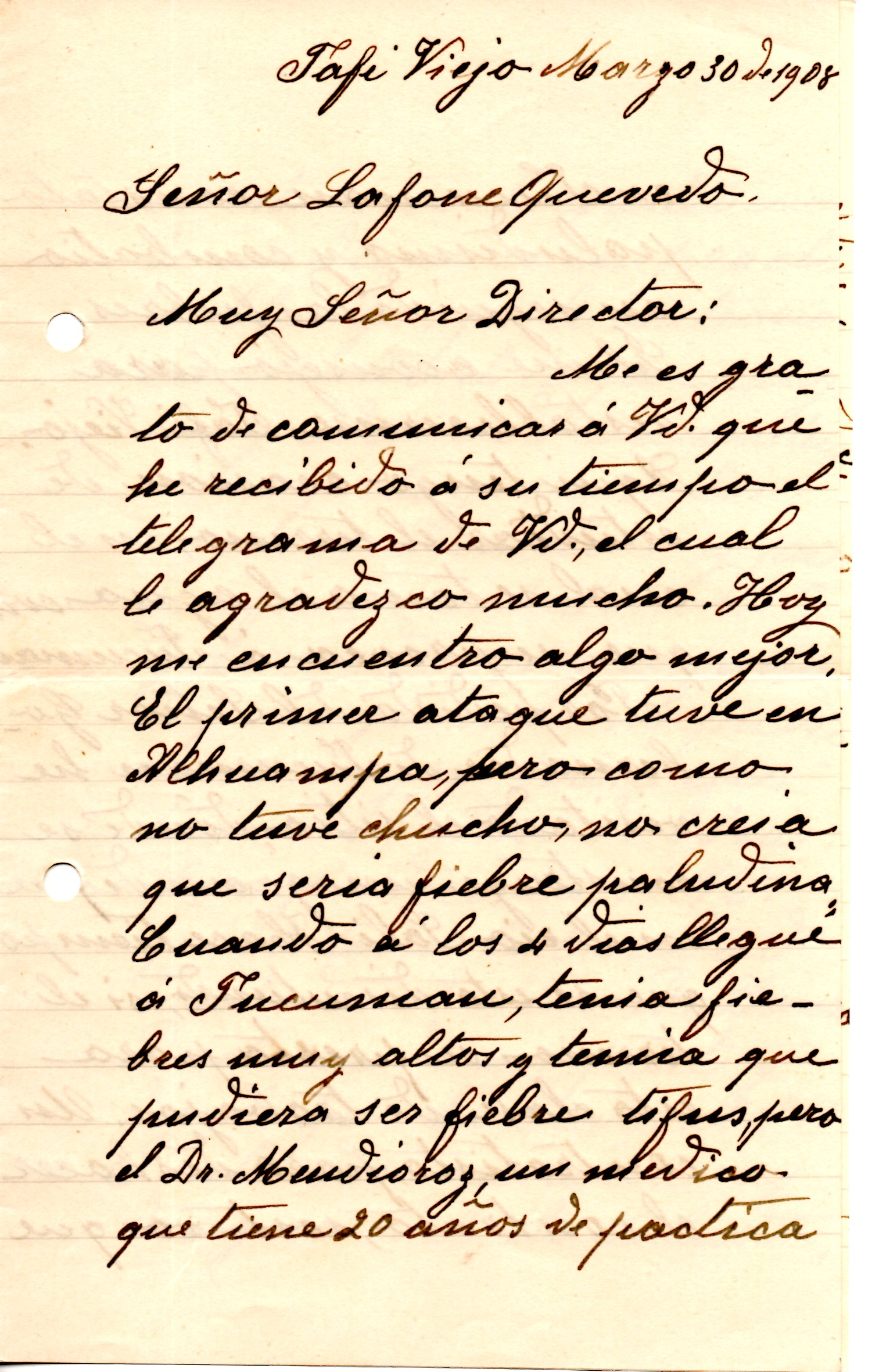


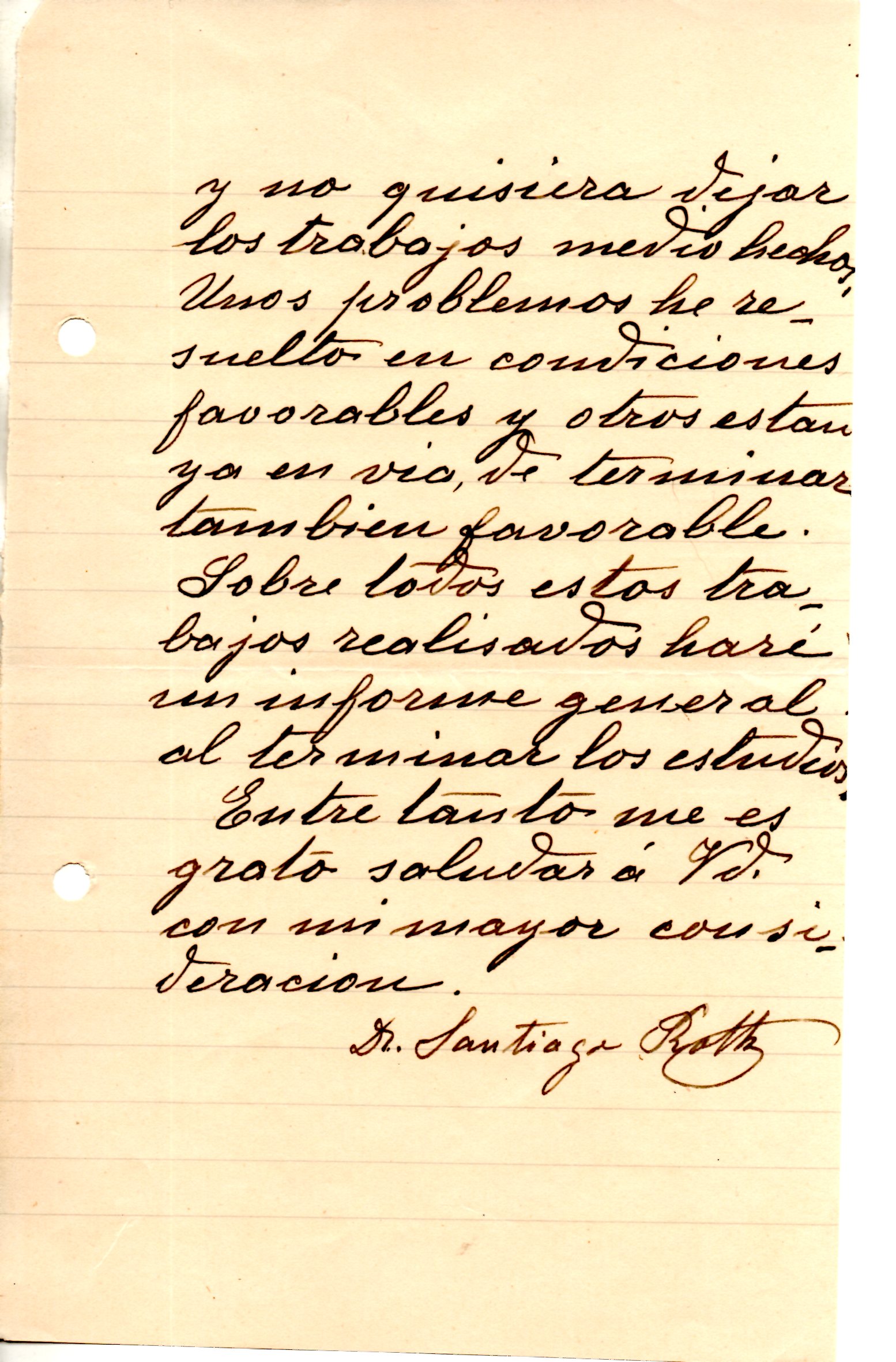

Supplement: Supplementary file 5 — Additional file 5. Letter of 1908 in which Santiago Roth informed the Director of the Museum de La Plata that he was recovering from malaria in Tucumán and about his hydrological works searching for drinkable water for the region. [file 13358_2023_282_MOESM5_ESM.docx]
