# Supplementary material for: From fossil trader to paleontologist: on Swiss-born naturalist Santiago Roth and his scientific contributions
Source: Swiss J Palaeontol. 2023 Sep 11;142(1):19. doi: 10.1186/s13358-023-00282-6 (PMC10495517; doi:10.1186/s13358-023-00282-6)
Supplement: Supplementary file 6 — Additional file 6. Transcription of the Letter from Santiago Roth to Hans Georg Stehlin in Basel inviting him to be his successor at the Museo de La Plata. [file 13358_2023_282_MOESM6_ESM.docx]

**Additional information**

**From fossil trader to palaeontologist: On Swiss-born naturalist Santiago Roth and his scientific contributions**

Marcelo R. Sánchez-Villagra, Mariano Bond, Marcelo Reguero, Tomás Bartoletti

**Contents**

1. Examples of fossil mammals collected by Santiago Roth, in the collections in Copenhagen and La Plata.

2. Estimates prices of Roth's fossils in Geneva by A. Dreyer. Supplementary Information

3. Title from the University of Zurich of Doctor Philosophiae Honoris Causa to Santiago Roth (1900).

4. Transcription of the letter by Santiago Roth to the President of the University of Zurich thanking him for the honorary doctorate title.

5. Letter of 1908 in which Roth informed the Director of the Museum de La Plata that he was recovering from malaria in Tucumán and about his hydrological works searching for drinkable water for the region in question.

6. Transcription of the Letter from Santiago Roth to Hans Georg Stehlin in Basel inviting him to be his successor at the Museo de La Plata.

**Additional Information 6.** Transcription of the Letter from Santiago Roth to Hans Georg Stehlin in Basel inviting him to be his successor at the Museo de La Plata.

Sehr geehrter Herr Dr. Stehlin!

Ich erlaube mir, mich in einer confidentiellen Angelegenheit an Sie zu wenden.

Seit längerer Zeit befinde ich mich gesundheitlich nicht gut und da ich bald 74 Jahre alt bin, so muss ich mich um einen Nachfolger umsehen.

Es wird Ihnen nicht unbekannt sein, dass das Museum von La Plata eine der grössten fossilen Säugetiersammlungen besitzt. Einen grossen Teil des Materials habe ich persönlich gesammelt und mit genauen Fundangaben versehen. Mein Wunsch geht dahin, dass die Sachen in solche Hände fallen möchten, dass sie im Sinne von Rütimeyer, d.h. stamgeschichtlich, bearbeitet würden. Ameghino hat allerdings die Säugetiere einigermassen filogenetisch bearbeitet, die Gebisse aber nicht dargestellt, wie sie in Wirklichkeit sind, sondern wie sie nach seiner Auffassung sein sollten, so dass alle Säugetiere von Typen aus Patagonien abstammen würden.

Schon zu Lebzeiten Rütimeyers hatte ich mit vergleichenden Studien über die Zahnentwicklung der Ungulaten, Notoungulaten und Primaten begonnen und stand diesbezüglich mit Rütimeyer und später mit Dr. Rud. Burckhardt im Briefwechsel, die Arbeit ist aber bis jetzt nicht fertig geworden. Da ich diese in Deutsch und Spanisch geschrieben habe, kann sie nicht so gedruckt werden, ein Fachmann wird sich aber nach den Fotografien und dem Geschriebenen schon zurecht finden.

Ich kenne Ihre Arbeiten und erlaube mir, Sie zu fragen, ob Sie eventuell geneigt wären, meine Stelle als Chef der palaeontologischen Abteilung am Museum in La Plata zu übernehmen. Das kann ich Ihnen versichern, dass Sie kaum anderswo ein solches Material zur Bearbeitung vorfinden werden. Ausserdem stehen auch die reichhaltigen Sammlungen vom Nationalmuseum in Buenos Aires und von Ameghino zur Verfügung.

Ich kenne Ihre Verhältnisse in Basel nicht und wenn Sie Gründe haben, die Sie dort festhalten, so können Sie mich vielleicht auf einen anderen für die Stelle geeigneten Fachmann aufmerksam machen.

Gerne einen geneigten Antwort entgegensehend, verbleibe ich mit ausgezeichneter Hochachtung.

Ihr ergebener

Dr. Santiago Roth

Adresse: S. Roth

calle 49 No. 922

La Plata Provincia Buenos Aires
